# Development and Validation of a Combined Hypoxia and Immune Prognostic Classifier for Lung Adenocarcinoma

**DOI:** 10.7150/jca.70725

**Published:** 2022-05-16

**Authors:** Hua Huang, Guangsheng Zhu, Ruifeng Shi, Yongwen Li, Zihe Zhang, Songlin Xu, Chen Chen, Peijun Cao, Zhenhua Pan, Hongbing Zhang, Minghui Liu, Hongyu Liu, Jun Chen

**Affiliations:** 1 Department of Lung Cancer Surgery, Tianjin Medical University General Hospital, Tianjin 300052, People's Republic of China.; 2 Tianjin Key Laboratory of Lung Cancer Metastasis and Tumor Microenvironment, Tianjin Lung Cancer Institute, Tianjin Medical University General Hospital, Tianjin 300052, People's Republic of China.; 3 Quantitative Biomedical Research Center, Department of Population and Data Sciences, University of Texas Southwestern Medical Center, Dallas, TX, USA, 75390.; 4 Department of Thoracic Surgery, First Affiliated Hospital, School of Medicine, Shihezi University, Shihezi, Xinjiang 832008, People's Republic of China.

**Keywords:** lung adenocarcinoma, hypoxia, immune, prognosis, immunotherapy

## Abstract

Lung cancer is the leading cause of cancer-related deaths worldwide. Hypoxia is a crucial microenvironmental factor in lung adenocarcinoma (LUAD). However, the prognostic value based on hypoxia and immune in LUAD remains to be further clarified. The hypoxia-related genes (HRGs) and immune-related genes (IRGs) were downloaded from the public database. The RNA-seq expression and matched complete clinical data for LUAD were retrieved from The Cancer Genome Atlas (TCGA) and Gene Expression Omnibus (GEO) database. The least absolute shrinkage and selection operator (LASSO) Cox regression analysis was applied to model construction. Hypoxia expression profiles, immune cell infiltration, functional enrichment analysis, Tumor Immune Dysfunction and Exclusion (TIDE) score and the somatic mutation status were analyzed and compared based on the model. Moreover, immunofluorescence (IF) staining in human LUAD cases to explore the expression of hypoxia marker and immune checkpoint. A prognostic model of 9 genes was established, which can divide patients into two subgroups. There were obvious differences in hypoxia and immune characteristics in the two groups, the group with high-risk score value showed significantly high expression of hypoxia genes and programmed death ligand-1 (PD-L1), and maybe more sensitive to immunotherapy. Patients in the high-risk group had shorter overall survival (OS). This model has a good predictive value for the prognosis of LUAD. We constructed a new HRGs and IRGs model for prognostic prediction of LUAD. This model may benefit future immunotherapy for LUAD.

## Introduction

Lung cancer is the most common malignancy and the leading cause of global cancer-related mortality 1, 2. Non-small cell lung cancer (NSCLC) accounts for approximately 85% of lung cancers and is the most numerous histological type 3. Despite considerable progress and advance in clinical diagnosis and multimodality treatments, the 5-year OS of patients with advanced lung cancer remains low 4, and LUAD is the most abundant subtype of NSCLC. The current treatment for LUAD is so limited that the development of effective therapies is urgent.

The immune evasion strategy is 1 of the 10 putative cancer hallmarks 5. Immune checkpoint blockade therapy has obvious advantages such as applicability to a wide range of cancer types and long-term clinical response 6, 7. The inhibitors of PD-L1 and CTLA-4 have shown good efficacy in the treatment of NSCLC 8. Some other checkpoint blockers have also been proven to improve the prognosis of NSCLC 9, 10. The major bottleneck of immunotherapy is only beneficial to a fraction of LUAD patients, and reliable prognostic immune markers for treatment effect evaluation are indispensable. Different models for predicting the prognosis of LUAD based on the tumor immunology 11 or tumor microenvironment have been established 12, but only a few models combine them for analysis, such as immunity and hypoxia.

Hypoxia is an important microenvironmental factor in LUAD, which drives the proliferation, metastasis, invasion and other aggressive characteristics of many tumors 13, 14. Hypoxic foci are formed when the metabolic demand of cancer cells exceeds the oxygen supply in the tumor blood vessels. Hypoxia-inducible factor is activated and mediates angiogenesis, epithelial-mesenchymal transition and metastasis 15. A previous study indicated that hypoxia is a vital factor leading to metabolic reprogramming, resistance to targeted therapies and poor OS in LUAD patients 16. Meanwhile, increasing evidence has demonstrated close interaction between hypoxia and immunity in LUAD 17. However, the specific mechanisms between them remain fuzzy, and few studies have explored all the HRGs and IRGs and their pathways enrichment in LUAD.

In the current study, through bioinformatics, we constructed a model based on HRGs and IRGs to analyze and explore the prognosis of LUAD patients. We demonstrated that the risk score is strongly linked to hypoxia and immune. We further used quantitative reverse transcription-polymerase chain reaction (qRT-PCR) and IF techniques to verify part of the results in tumor tissue samples from the Tianjin Medical University General Hospital (TJMUGH) cohort.

## Materials and Methods

### Data acquisition

**Figure 1:**
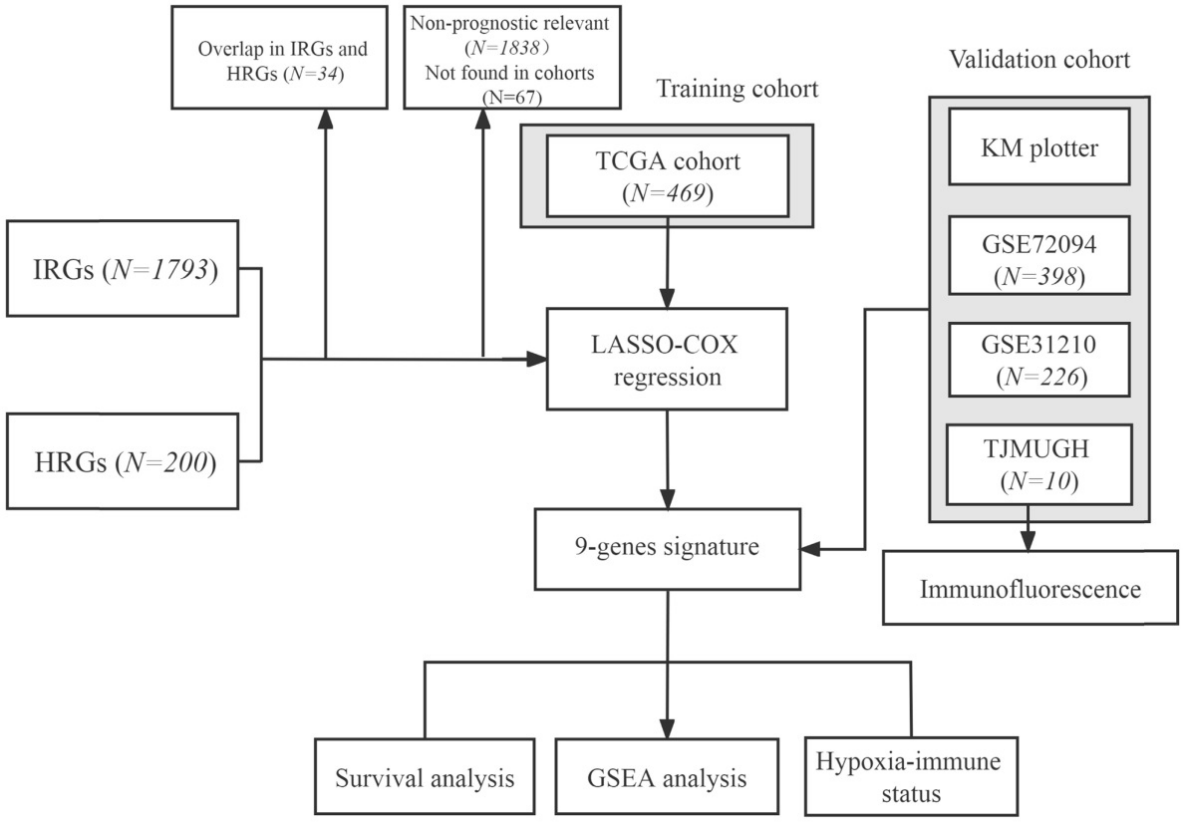
Flowchart of this study.

The flowchart of this research is shown in Figure 1. The level 3 RNA sequencing data and matched complete clinical information of 469 patients with LUAD were retrieved from TCGA (https://portal.gdc.cancer.gov/repository), hereafter referred to as the TCGA cohort. The microarray expression profile datasets GSE31210 and GSE72094 were obtained from the GEO (http://www.ncbi.nlm.nih.gov/geo/); GSE31210 contained 226 LUAD samples and hereafter referred to as the GSE31210 cohort. GSE72094 contained 398 LUAD samples and hereafter referred to as the GSE72094 cohort. The detailed information was provided in Table S1. Patients who met the following selection criteria were included: (a) histologically diagnosed with LUAD; (b) available gene expression data; (c) available survival and clinical information.

### Generation of HRGs and IRGs

The list of HRGs was obtained from the hallmark gene sets of the Molecular Signatures Database (https://www.gsea-msigdb.org/gsea/msigdb/), including 200 genes and was supplemented in Table S2. The list of IRGs was downloaded from ImmPort (https://www.immport.org), including 1793 genes and was supplemented in Table S3. A total of 200 HRGs and 1793 IRGs were analyzed in all cohorts.

### Construction and validation of a prognostic HRG and IRG signature

HRGs and IRGs first were performed univariate Cox regression in TCGA and GSE31210, respectively. So that prognostic-related genes can be screened. Then, we performed the LASSO Cox regression analysis to construct a prognostic model in the TCGA cohort. “Glmnet” package was carried out for LASSO Cox regression. The risk score of each patient was calculated based on a unified formula. The formula was constructed as follows: risk score = 0.007277 × ADM + (-0.08605) × ARRB1 + 0.014984 × DDIT4 + 0.132006 × ERO1A + 0.066597 × FURIN + 0.181954 × LDHA + (-0.04757) × NAGK + 0.077204 × NR2F2 + 0.001257 × NRAS. The GSE31210 and GSE72094 cohorts were used for verification.

### Estimation of immune cell type fractions

xCell (https://xcell.ucsf.edu) is a web tool, which can estimate the abundance scores of 64 immune and stromal cell types fractions 18. xCell was applied to calculate the proportion of immune and stromal cells in the TCGA and GSE31210 cohorts, and the microenvironment and stromal scores were also calculated. CIBERSORT (https://cibersort.stanford.edu/) is an analysis tool to estimate the abundance of 22 immune member cell types 19.

### Gene Set Enrichment Analysis (GSEA) and tumor mutation burden (TMB) analysis

GSEA was applied to elucidate relevant signaling pathways 20, using the “Clusterprofler” package in R software. The inclusion criteria were as follows: normalized enrichment score > 1, and false discovery rate q-value < 0.25. The somatic mutation data was extracted from the GDC database portal (https://portal.gdc.cancer.gov/), the “maftools” R package was conducted to perform the visualization process 21.

### RNA extraction and qRT-PCR analysis

We collected 10 patients with LUAD at TJMUGH from 2018 to 2019, the samples came from surgery, hereafter referred to as the TJMUGH cohort. In the TJMUGH cohort, total RNA was extracted from the 10 samples by TRIzol reagent (Invitrogen, MA, USA), and PrimeScript RT Reagent Kit (TaKaRa, Dalian, China) was used to synthesize cDNA according to the manufacturer's instructions. Using β-actin mRNA as an internal control to normalize the expression levels of 9 genes. The primers used in this study were supplemented in Table S4, and the normalized expression levels of 9 genes were supplemented in Table S5.

### Immunofluorescence

IF assays were conducted as previously described 22. The formalin-fixed paraffin-embedded sections were deparaffinized and rehydrated, EDTA antigen retrieval buffer was used for antigen retrieval at 98 °C for 8 min. Then the slides were soaked in 3% hydrogen peroxide for 15 minutes. Anti-carbonic anhydrase 9 (CA-IX) (Servicebio, Wuhan, Hubei, China) and PD-L1 (Servicebio, Wuhan, Hubei, China) were applied at 4 °C overnight. Then, Alexa Fluor-conjugated secondary antibodies were used. Digital slide scanner (Pannoramic 250, 3DHistech, Hungary) were performed to scan samples. Data are expressed as mean ± SD and analyzed by Student's t-test.

### Immunotherapeutic response prediction

TIDE is a simulation method for calculating tumor immune escape through the two main mechanisms and would be used for immunotherapy of cancer patients with response analysis 23. The TIDE score of each patient was calculated through the online web tool TIDE (http://tide.dfci.harvard.edu/), and then the scores were compared in the two groups.

### Statistical analysis

PCA analysis was carried out using the “prcomp” function of the “stats” R package. The OS was compared using the Kaplan-Meier analysis with the log-rank test. The time-dependent ROC curve was calculated using the “timeROC” R package. A* P*-value < 0.05 was considered statistically significant.

## Results

### Overview of hypoxia and immune signatures

**Figure 2:**
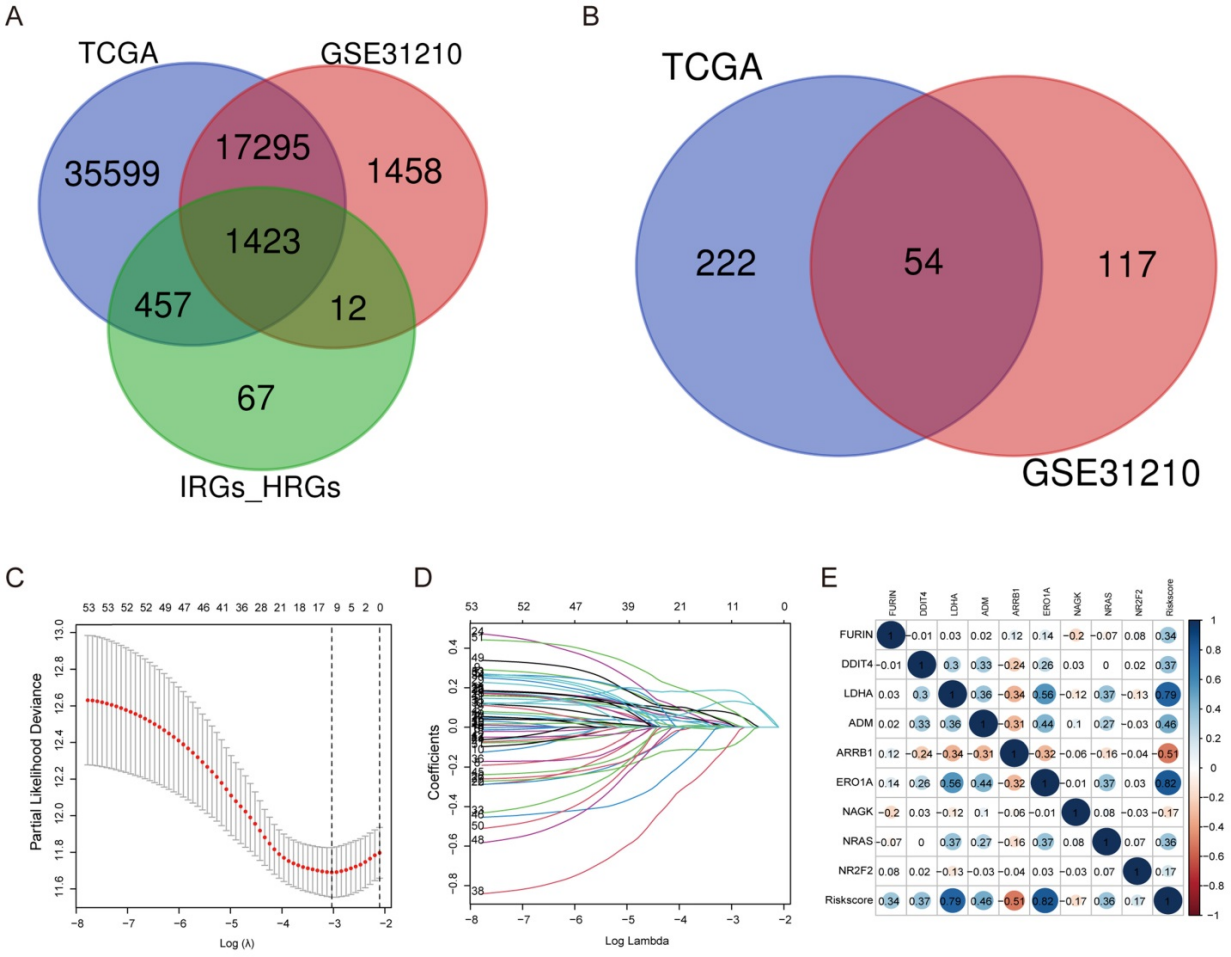
** Construction of a predictive model of LUAD. (A)** Venn diagram showing 1423 genes identified in the TCGA and GSE31210 cohorts. **(B)** Venn diagram showing 54 prognostic associated genes identified in the TCGA and GSE31210 cohorts. **(C, D)** Screening the optimal parameter (lambda), which is represented by the vertical black line in the plot. **(E)** Correlation between the risk score value and the nine genes.

A total of 200 HRGs and 1793 IRGs were used to construct the prognosis model. Thirty-four were removed because they were identified as both hypoxia and immune genes (Figure 2A). We conducted univariate analysis of OS to obtain genes of prognostic values in the TCGA and GSE31210 cohorts, respectively. The results indicated that 54 genes had a close relationship with OS in both the GSE31210 and TCGA cohorts (Figure 2B).

### Construction of the hypoxia and immune gene prognostic model in LUAD

**Figure 3:**
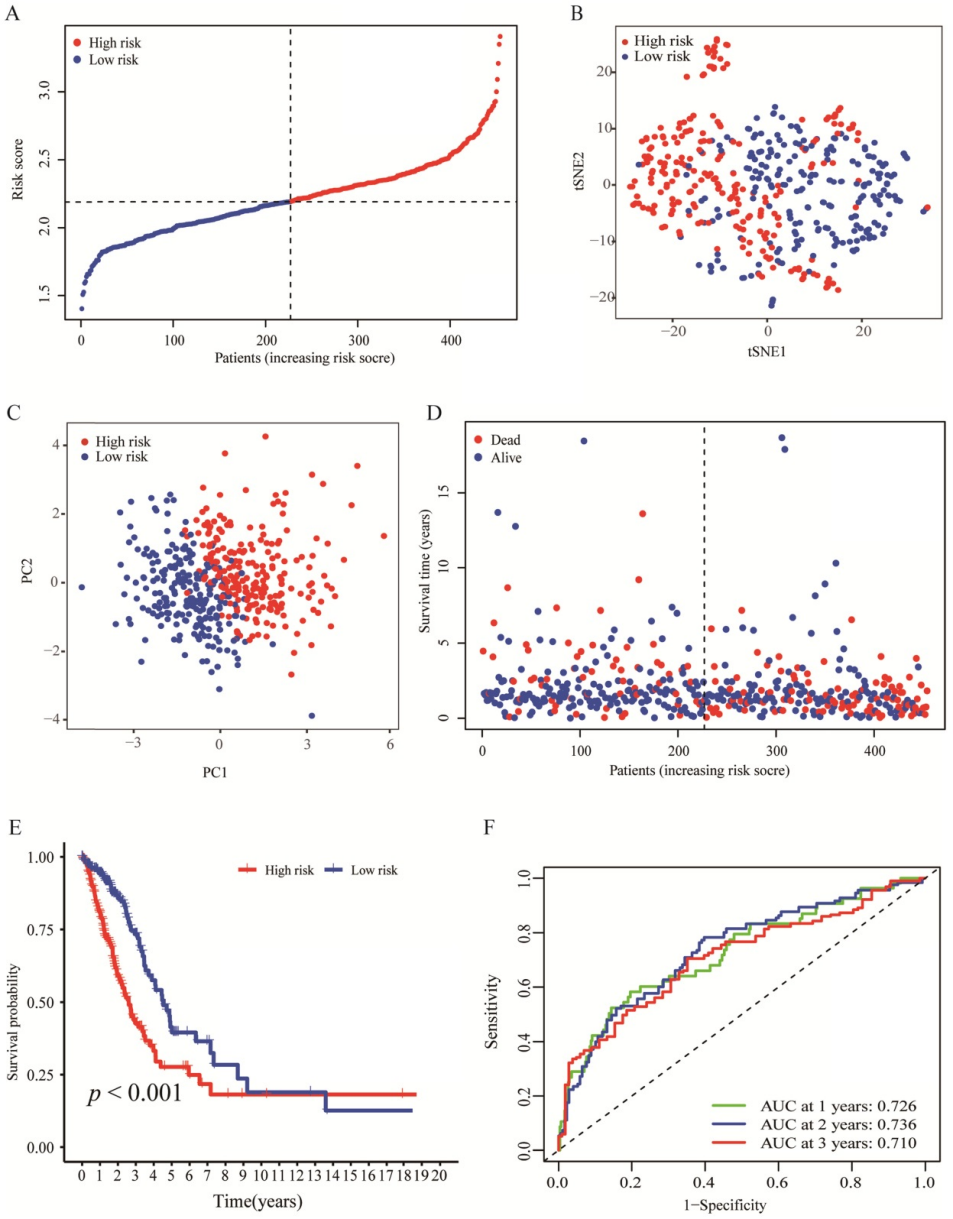
** Evaluation of the nine-genes signature in the TCGA cohort. (A)** The distributions of the risk score. **(B, C)** t-SNE and PCA analysis showed significant differences between groups of patients. **(D, E)** The distributions of OS status and OS of patients between high-risk and low-risk groups, patients in the high-risk group had higher score values and mortality. **(F)** Time-independent ROC analysis of the risk score for prediction of the OS, the area under the curve for 1, 2, and 3 years reached 0.726, 0.736, and 0.710, respectively.

By making use of the expression data of 54 genes in the TCGA cohort, we conducted the LASSO Cox regression to construct a prognostic model for the OS in LUAD patients. A nine-gene signature was selected through the partial likelihood deviance method. The results are shown in Figures 2C, D. Among them, DDIT4, LDHA, ERO1A, and NAGK are HRGs, whereas FURIN, ARRB1, NRAS, and NR2F2 are IRGs, and ADM participated in both hypoxia and immune process. The individual prognostic value of each gene is further verified in the Kaplan-Meier plotter database (Figures S1A-I). According to the above formula, we obtained the risk score of each patient. Pearson correlation analysis showing the association of risk score values with 9 genes (Figure 2E). On the grounds of the median cut-off value of the risk score, we separated patients into low-risk or high-risk groups (Figure 3A). t-SNE and PCA analysis we conducted manifested that different groups of patients were distinctively clustered (Figures 3B, C). Patients in the high-risk group are more likely to die early than those in the low-risk group (Figure 3D). Through the survival analysis, we obtained a marked difference between the two subgroups, the OS of the high-risk group was dramatically lower (Figure 3E;* P* < 0.001). Using the R software to draw the time-dependent ROC curve, the result showed that the area under the curve (AUC) for 1, 2, 3 years reached 0.726, 0.736, 0.710, respectively (Figure 3F). The ROC curve plot demonstrated that this model has excellent predictive value for short-term and long-term follow-up.

### Validation of the gene signature in LUAD

**Figure 4:**
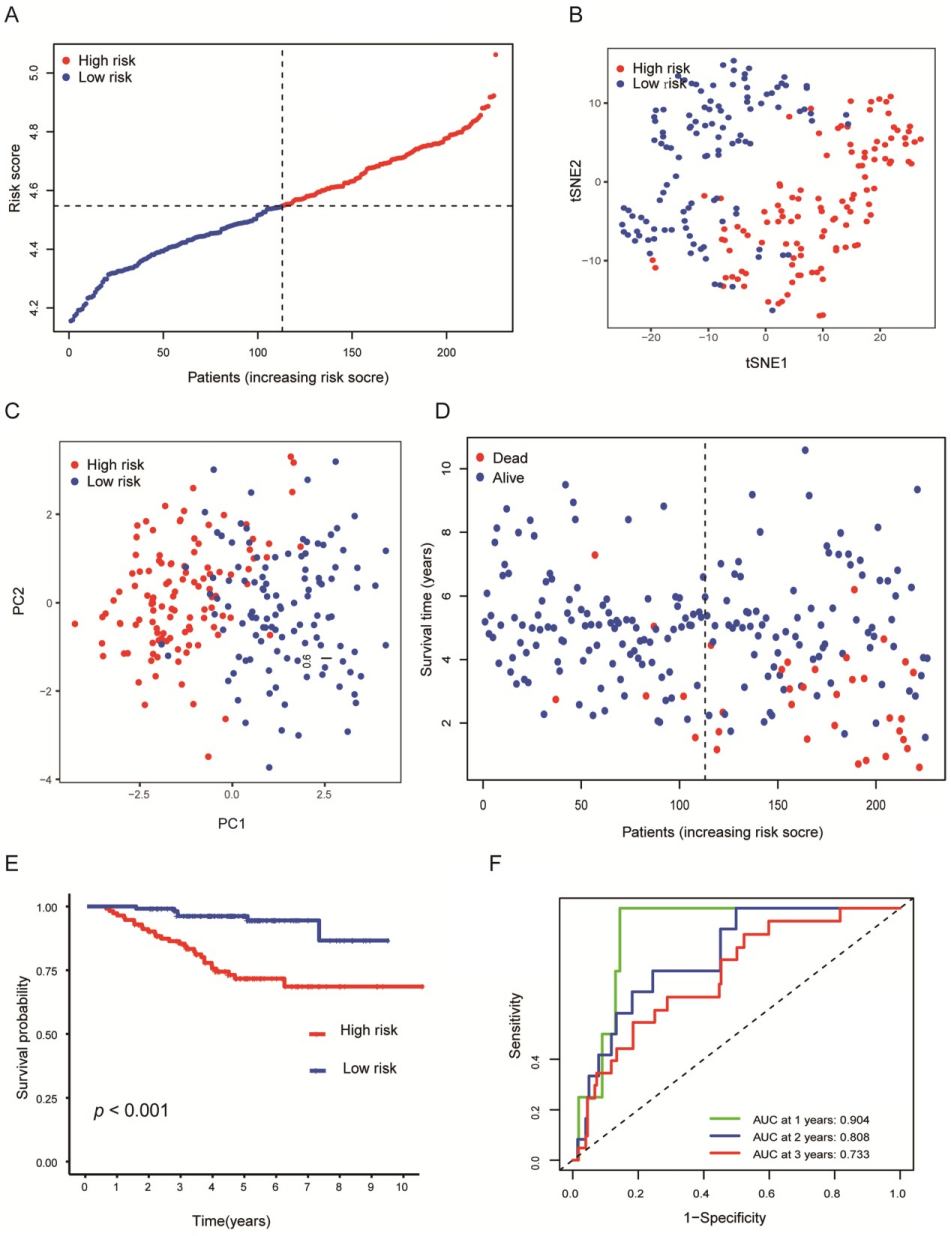
** Validation of the nine-genes signature in the GSE31210 cohort. (A)** The distributions of the risk score. **(B, C)** t-SNE and PCA analysis showed significant differences between groups of patients. **(D, E)** The distributions of OS status and OS of patients between high-risk and low-risk groups, patients in the high-risk group had higher score values and mortality. **(F)** Time-independent ROC analysis of the risk score for prediction of the OS, the area under the curve for 1, 2, and 3 years reached 0.904, 0.808, and 0.733, respectively.

To verify the stability of the predicted prognosis of the model, we also used the above formula to calculate the risk score of patients in the GSE31210 and GSE72094 cohorts. In the GSE31210 cohort, t-SNE and PCA analysis demonstrated that the patients were distinctively clustered by the median value of the risk score (Figures 4A-C). Likewise, the OS of patients in the high-risk group was dramatically shorter (Figures 4D, E; *P* < 0.001), and the AUC for 1, 2, 3 years reached 0.904, 0.808, 0.733, respectively (Figure 4F), which indicated that this model has excellent predictive value for short-term follow-up. In the GSE72094 cohort, the same conclusion was also obtained (Figures S2A-F).

### Hypoxia profiling

**Figure 5:**
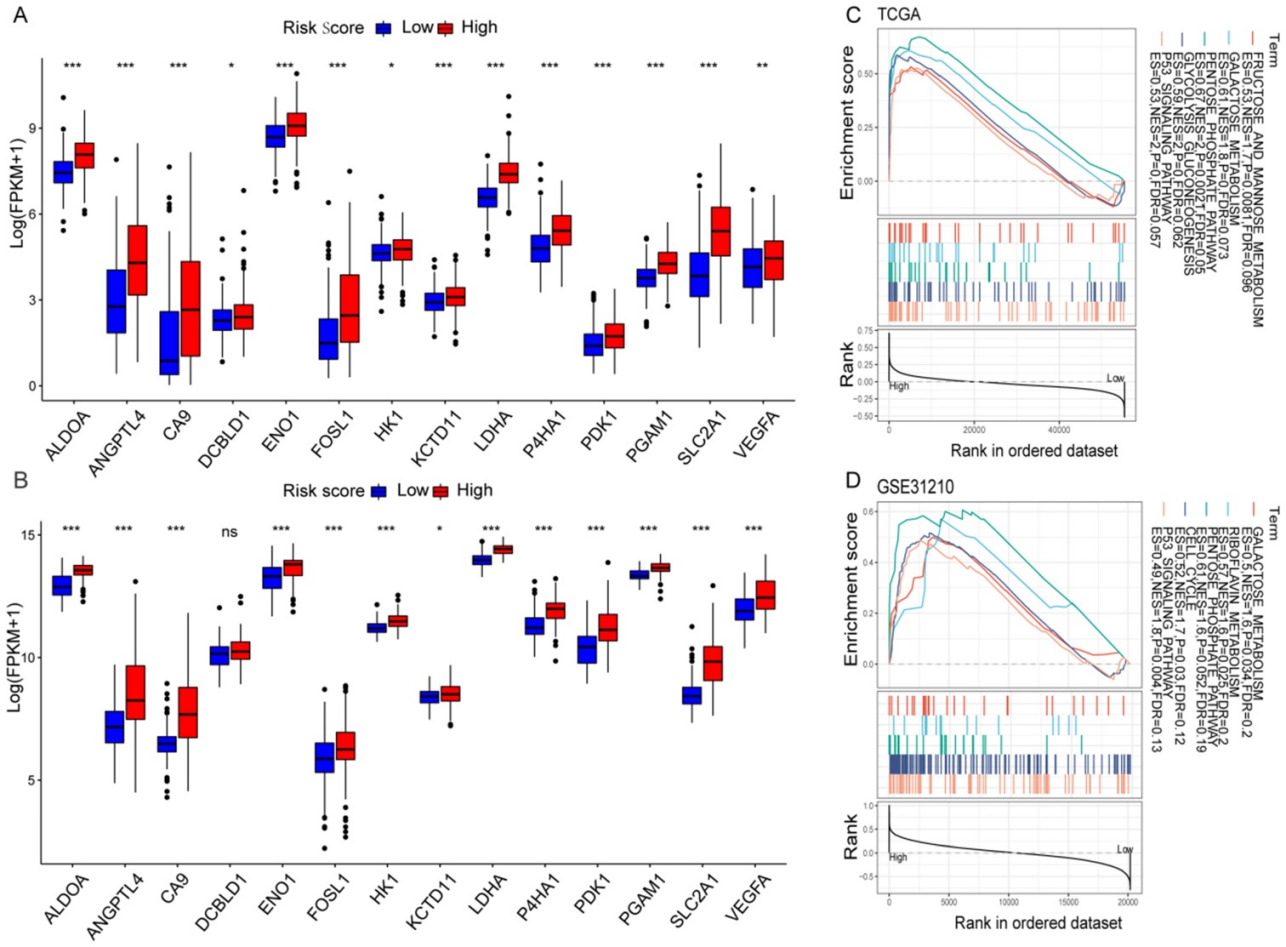
** Hypoxia profiles in the risk score stratified groups. (A, B)** Box plot showed that the expression of key hypoxia genes was significantly higher in the high-risk group of the TCGA (A) and GSE31210 (B) cohorts. **(C, D)** GSEA demonstrated that hypoxia-related biological processes enriched in the high-risk group of the TCGA (C) and GSE31210 (D) cohorts. *P < 0.05, **P < 0.01, ***P < 0.001, and ns *P* > 0.05.

The correlation between the risk score values and the selected HRGs indicated that the risk score might reflect hypoxia status in LUAD. Moreover, according to the previous studies of key hypoxia genes in cancer 24, the changes of 14 key hypoxia marker genes at the transcriptome level were compared between the high-risk and low-risk groups. The results revealed that among the high-risk populations in the TCGA cohort, the selected hypoxia marker genes were dramatically up-regulated (Figure 5A). The same verification was also carried out in the GSE31210 cohort. Except that there was no difference in the DCBLD1, the other changes were completely consistent with the TCGA cohort (Figure 5B). It was found that ALDOA has been identified as an important factor for cancer cell proliferation 25. The activation of LDHA may become a new therapeutic target due to it promotes the invasion and metastasis of cancer cells 26, and VEGFA is the main driver of angiogenesis and vascular permeability 27. The results indicated that the malignant transformation of tumors induced by hypoxia may be more prevalent in high-risk populations. To elucidate relevant biological signal pathways involved in high-risk patients, GSEA was further applied to the TCGA and GSE31210 cohorts. The results demonstrated that in the high-risk group of the TCGA cohort, hypoxia-related biological pathways were dramatically enriched (Figure 5C). Interestingly, similar hypoxia-related pathways also exist in the GSE31210 cohort (Figure 5D). All these results indicated a high hypoxia status in the high-risk group.

### Immune profiling

**Figure 6:**
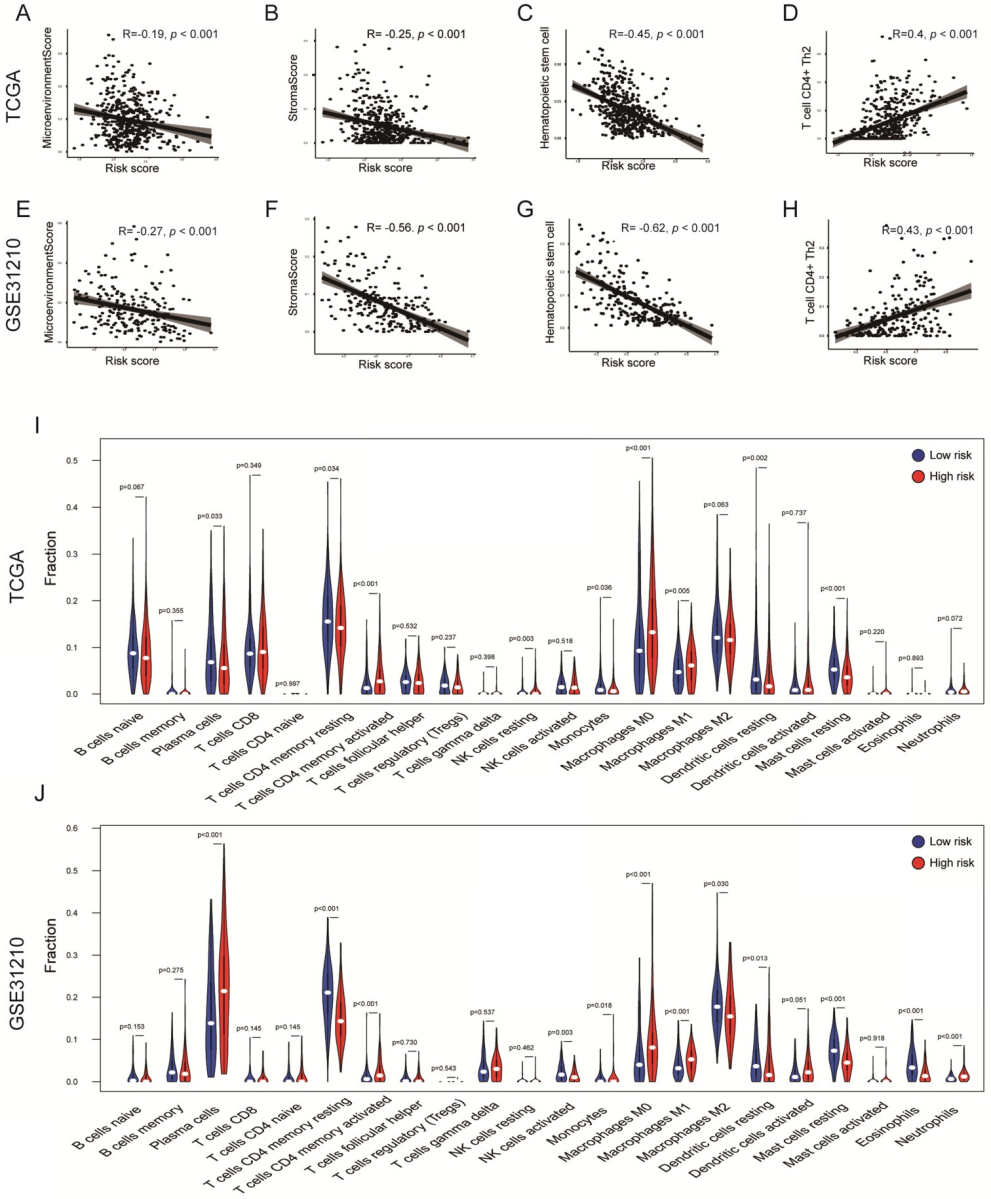
** Immune profiles in the risk score stratified groups. (A-H)** Correlation analysis of the risk scores value and microenvironment scores (A, E), stromal scores (B, F), hematopoietic stem cells (C, G), T cell CD4+ Th2 (D, H) in the cohorts. Spearman's rank correlation analysis was used for data analysis. **(I, J)** The comparison of immune cell fractions between groups of the TCGA (I) and GSE31210 (J) cohorts via the CIBERSORT method. The Wilcoxon signed-rank test was used for data analysis.

To explore whether the risk score can reflect the immune microenvironment of LUAD, xCell was applied to estimate the immune cell fractions in the TCGA and GSE31210 cohorts. Moreover, we calculated the microenvironment and stromal scores of each patient. The risk score value was inversely correlated with the microenvironment score in the TCGA cohort (R = -0.19, *P* < 0.001); a similar result was confirmed in the GSE31210 cohort (R = -0.27, *P* < 0.001; Figures 6A, E) . This result showed that the risk score may have an impact on the tumor microenvironment (TME). Interestingly, the risk score was also inversely correlated with the stromal score (TCGA: R = -0.25, *P* < 0.001; GSE31210: R = -0.56, *P* < 0.001; Figures 6B, F), indicating that risk score might also be associated with fibroblasts. Hematopoietic stem cells are the source of immune cells 28, whereas T cell CD4 Th2 is responsible for the inhibition of several macrophage functions 29. A significant negative correlation between risk score and hematopoietic stem cells was observed in two independent cohorts (TCGA: R = -0.45, *P* < 0.001; GSE31210: R = -0.62, *P* < 0.001; Figures 6C, G). However, the risk score was positively correlated with T cell CD4 Th2 in the TCGA and GSE31210 cohorts (TCGA: R = 0.4, *P* < 0.001; GSE31210: R = 0.43, *P* < 0.001; Figures 6D, H). Furthermore, through the CIBERSORT algorithm, we found that tumor-infiltration immune cells (TICs) were significantly different between the two groups. T cell CD4 memory activated, Macrophages M1 and M0 were all significantly up-regulated in the high-risk groups of the TCGA and GSE31210 cohorts. Dendritic cells resting, Mast cells resting and T cell CD4 memory resting were all significantly down-regulated in high-risk groups of the TCGA and GSE31210 cohorts. In addition, plasma cells (*P* = 0.033) and monocytes (*P* = 0.036) were less abundant in the high-risk group in the TCGA cohort, whereas the opposite trend was seen in the GSE31210 cohort. NK cell resting was only more abundant in the high-risk group in the TCGA cohort (*P* = 0.003), and was unchanged in the GSE31210 cohort. In the GSE31210 cohort, NK cell activated (*P* = 0.003) and Eosinophils (*P* < 0.001) were lower in the high-risk group, and Neutrophils were higher in the high-risk group (*P* < 0.001). These results further supported the impact of risk scores on TME immune activity (Figures 6I, J).

### High-risk group expresses higher PD-L1 and hypoxia marker and is more sensitive to immunotherapy

**Figure 7:**
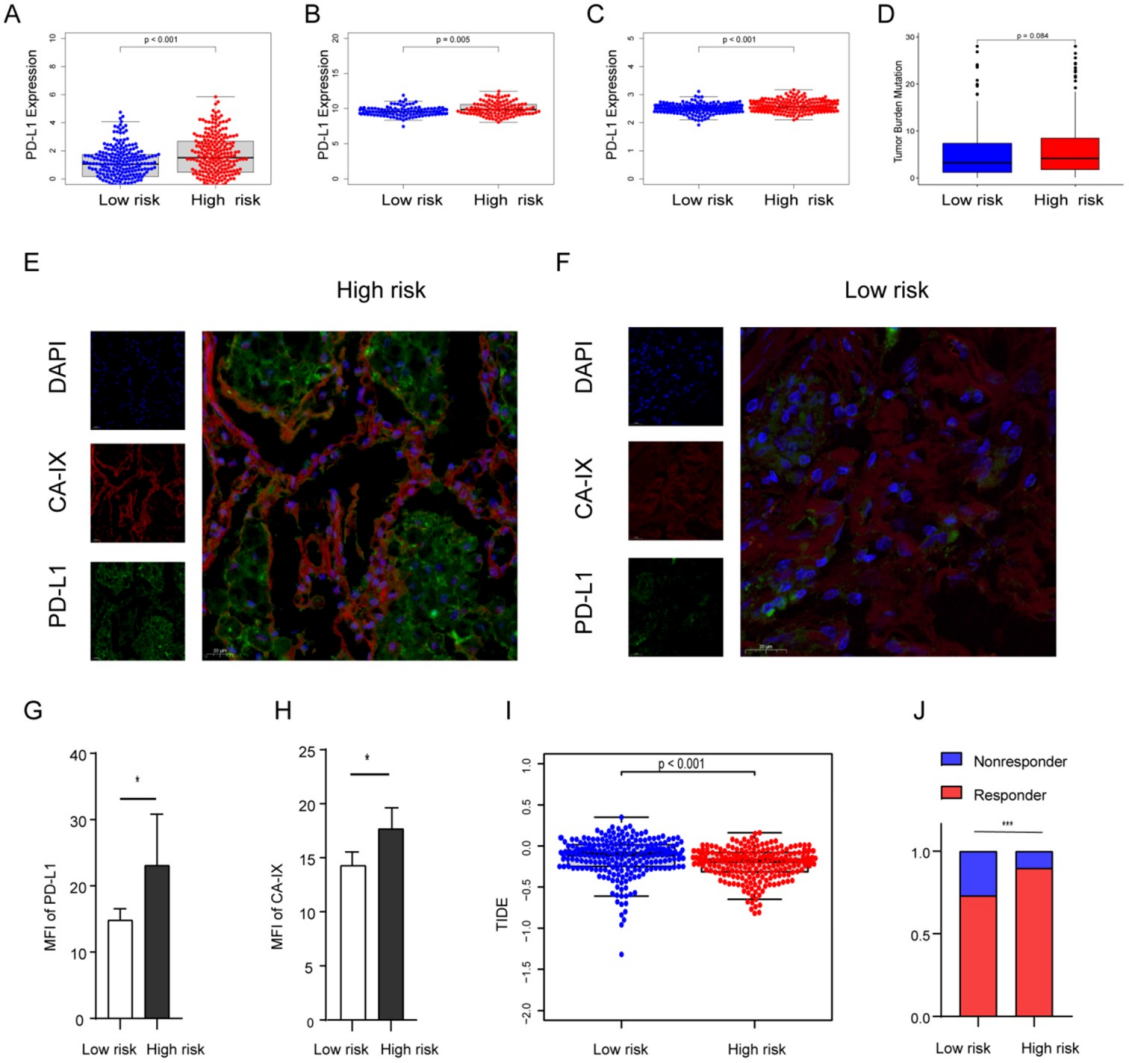
** The expression level of PD-L1, TMB, IF, and TIDE score in the risk score stratified groups. (A, B, C)** PD-L1 is more expressed in high-risk groups in the TCGA (A), GSE31210 (B) and GSE72094 (C) cohorts. **(D)** TMB was higher in the high-risk group in the TCGA cohort (*P* = 0.084). **(E, F)** Representative images of using IF to detect CA-IX (red), PD-L1 (green) and DAPI (blue) in the TJMUGH cohort. **(G, H)** Statistical analysis shows the differences in the mean fluorescence intensity (MFI) of PD-L1 (G) and CA-IX (H) between groups, the expression of PD-L1 and CA-IX was higher in the high-risk patients. **(I, J)** The differences of TIDE score (I) and immunotherapy sensitivity (J) between groups, high-risk group have lower TIDE scores and higher response rates in the TCGA cohort. *P < 0.05, ***P < 0.001.

Previous evidence has demonstrated that the relationship between hypoxia and PD-L1, and PD-L1 is a predictive biomarker for the efficacy of immunotherapy and contributes to disease progression with evasion from tumor immunity. The expression difference of PD-L1 was compared in the two groups. Our results showed that PD-L1 in the high-risk group is highly expressed in all cohorts (Figures 7A-C). To further confirm the results, we validated them in the TJMUGH cohort (N = 10). We performed qRT-PCR to detect the expression of 9 genes and calculate the risk score according to the same formula. Increasing evidence has demonstrated that CA-IX is induced by hypoxia and can promote cancer progression. Functionally, it is closely related to acidosis, aggressiveness and treatment resistance. CA-IX was used as an indicator of tumor cell hypoxia status 30. Therefore, IF was used to examine the protein level of CA-IX and PD-L1 (Figures 7E-H). The result was the same as the above, IF staining in human LUAD cases demonstrated that both CA-IX and PD-L1 were overexpressed in the high-risk group (*P* < 0.05). To future explore the role of this model in predicting the response to immunotherapy, we performed TIDE algorithm to evaluate each patient of the TCGA cohort (*P* < 0.001; Figure 7I). The result showed that the TIDE score of the high-risk group was lower, which means that the high-risk group may be more sensitive to immunotherapy. Moreover, there was a significantly higher response rate of immunotherapy in the high-risk group (*P* < 0.001; Figure 7J).

The differences in genomic mutation were compared between the two groups. The top five mutations in the high-risk score subgroup were TP53 (48%), TTN (48%), MUC16 (39%), CSMD3 (36%), and RYR2 (36%), whereas those in the low-risk score subgroup were TP53 (43%), MUC16 (39%), TTN (38%), CSMD3 (33%), and RYR2 (32%) (Figures S3A, B). In summary, the TMB score is consistent with the above results, suggesting TMB is higher in the high-risk group (p = 0.084; Figure 7D), which suggests that hypoxia may be an important cause of gene mutation.

### Independent prognostic role of the risk score

**Figure 8:**
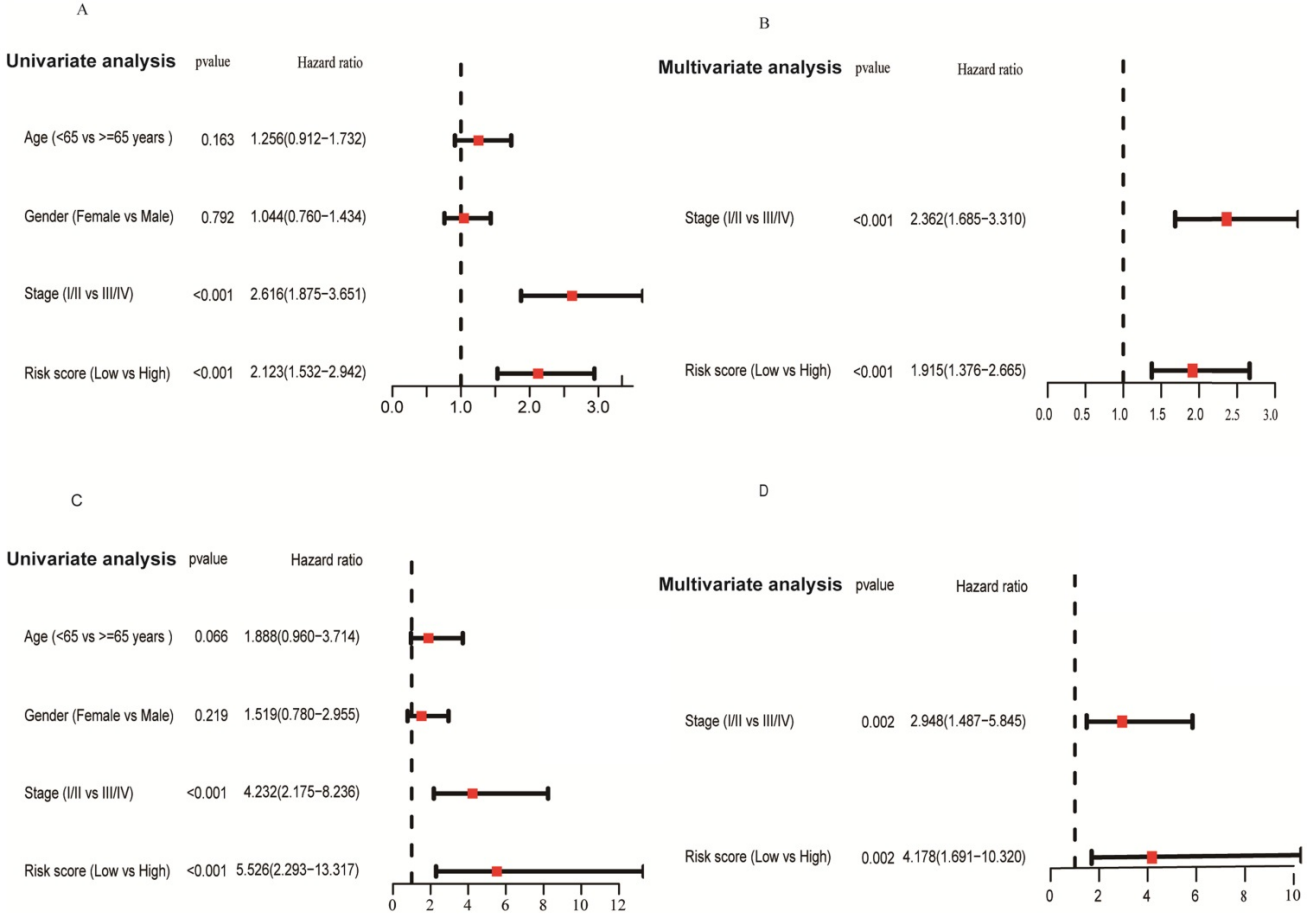
Forrest plot of the univariate and multivariate Cox regression analyses regarding OS in the TCGA **(A, B)** and the GSE31210 **(C, D)** cohorts.

We applied univariate and multivariate analysis to explore the predictive value of risk score for OS. The risk score value was significantly associated with OS in both the TCGA (HR = 2.123, 95% CI = 1.532-2.942, *P* < 0.001; Figure 8A) and GSE31210 cohorts (HR = 5.526, 95% CI = 2.293-13.317, *P* < 0.001; Figure 8C). After adjusting confounding factors, multivariate analysis demonstrated that the risk score remains an independent predictor for OS (TCGA cohort: HR = 1.915, 95% CI = 1.376-2.665, *P* < 0.001; GSE31210 cohort: HR = 4.178, 95% CI = 1.691-10.320, *P* = 0.002; Figures 8B, D).

## Discussion

In this study, a novel HRGs and IRGs prognostic model is constructed by using the expression data of the TCGA and GSE31210 cohorts. This model contains 9 key genes (FURIN, DDIT4, LDHA, ADM, ARRB1, ERO1A, NAGK, NRAS, and NR2F2). FURIN protein activation is functionally related to the increased aggressiveness of a variety of tumors and may be promising in targeted therapy 31. The activation of DDIT4 is related to the regulation of tumor mTOR signaling pathway, differentiation and expression of pluripotency gene 32. LDHA has been proven to directly increase the malignancy of tumors by regulating the production of reactive oxygen species and promoting lactic acid production. Moreover, it is used as a target for diagnosis in clinical trials 33. ADM is involved in immune regulation and trophoblast invasion under hypoxic conditions 34. ARRB1 has been demonstrated to be a potential tumor promoter in prostate cancer and is important for metabolic alterations in prostate cancer 35. ERO1A is significantly up-regulated, which predicts poor prognosis in LUAD 36, 37. We future investigated the prognostic prediction performance of this model. The survival time of patients in the low-risk group increased significantly. A recent study constructed a model of 2 immune-related genes to predict the prognosis of LUAD patients 11, the AUCs of the models for the two immune-related genes in the TCGA and GSE31210 cohorts were 0.706 and 0.680 at 1 year , respectively; in contrast, the AUC of the model in this study were 0.726 and 0.904 at 1 year, respectively, indicating that the model established in our study has slightly better predictability. In addition, this model was also more predictable at long-term follow-up. Although the prognostic model of LUAD established by hypoxia-related genes previously has similar predictive power to our study 38. However, this study combined HRGs and IRGs to construct a predictive model, which could more comprehensively reflect the hypoxia and immune status of LUAD patients.

The key hypoxia markers are higher in the high-risk group, which indicates that there are different hypoxia statuses in the tumor. Further, GSEA demonstrated that genes in the high-risk score group were remarkably enriched in the hypoxia-related pathway. These biological processes are closely related to hypoxia status, suggesting a high sensitivity of this model to predict hypoxia status in this subgroup.

Increasing focus has been attached to the impact of hypoxia on the tumor immune microenvironment, but the underlying correlation between tumor immunity and hypoxia is still unclear. xCell uncovered that the risk score value has an inverse correlation with the microenvironment score and stromal score. To further understand the underlying relationship between hypoxia status and immunity, our result indicated that the high-risk group had a higher fraction of T cell CD4 memory activated, macrophages M0 and M1. T cell CD4 memory preferentially reside in peripheral tissues, such as the skin, gut, and lung, allowing memory T cells to respond directly and rapidly to the presence of pathogens in peripheral tissues 39. It has been reported that hypoxia affects the lymphocyte expression of interleukin 2 and the antitumor function of T cells 40, 41, which may suggest that T cell CD4 memory is insensitive or less responsive to hypoxia in LUAD. In tumor tissues, tumor-associated macrophages are thought to differentiate into two main phenotypes: proinflammatory M1 and protumorigenic M2. It has been reported that over 80% of studies show a correlation between macrophage density and poor patient prognosis 42. We noticed that plasma cells and monocytes showed opposite trends in the TCGA and GSE31210 cohorts, and plasma cells, also known as effector B cells. The prognostic impact of the plasma cell marker CD138, which is expressed in epithelial tumor cells and other stromal cells, remains unclear. Tumor-infiltrating CD138+ plasma cells are associated with improved prognosis in NSCLC and colorectal cancer 43, 44, but poor prognosis in breast cancer and epithelial ovarian cancer 45, 46. Monocytes originate from progenitor cells in the bone marrow and are transported to peripheral tissues through the blood, where they can phagocytose and remove injured and senescent cells and their debris 47. For plasma cells and monocytes in the TCGA and GSE31210 cohorts showed different changes, we speculated that this may be due to the different clinical stages of the patients in the two cohorts, we noticed that all the patients in the GSE31210 cohort were patients with early-stage LUAD, Their relationship with prognosis is also worthy of further exploration. We found that PD-L1 expression levels were higher in patients in the high-risk group. PD-L1 is closely related to the immunotherapy effect of LUAD, reflecting the underlying immune efficacy in tumors. Previous evidence has confirmed that PD-L1 is up-regulated under hypoxic conditions in macrophages and myeloid-derived suppressor cells 48, 49. Another study has demonstrated that GBE1 inhibitors can recruit CD8+ T lymphocytes in lung cancer microenvironment, together with the up-regulation of PD-L1 50.

In our study, high-risk group patients of the TCGA and GSE31210 cohorts showed a higher expression of PD-L1. Moreover, IF showed that the expression levels of CA-IX and PD-L1 in the high-risk group were higher of the TJMUGH cohort. Consistent with the above results, TIDE analysis revealed that the high-risk group may be more sensitive to immunotherapy. The complex interaction between hypoxic environments and immune checkpoints remains to be further explored. However, we should note some deficiencies in our study. First, this is a retrospective, cross-cohort study based on public databases. Second, the TJMUGH cohort is small in size and lacks survival data. Third, only including the expression of PD-L1 for analysis cannot accurately define the impact of hypoxia on immune characteristics. Finally, the effect of this model on immunotherapy has not been accurately assessed due to the lack of external data. Hence, more research and data are needed to confirm the clinical application of our model.

Hypoxia acts a pivotal part in regulating tumor immune privilege or immunotherapy 51. High risk means a relatively high coefficient of the hypoxic microenvironment and immune checkpoint. These data indicate that our model may be beneficial for immunotherapy of LUAD and is worthy of further research.

In summary, we developed and validated a prognostic molecular classifier based on hypoxia and immune expression profiles. We hope that this classifier will be beneficial for immunotherapy and prognosis prediction.

## Supplementary Material

Supplementary figures and tables.
