# Supplementary material for: Development and Validation of a Combined Hypoxia and Immune Prognostic Classifier for Lung Adenocarcinoma
Source: J Cancer. 2022 May 16;13(8):2631–43. doi: 10.7150/jca.70725 (PMC9174870; doi:10.7150/jca.70725)
Supplement: Supplementary file 1 — Supplementary figures and tables. [file jcav13p2631s1.pdf]

# Supplementary Material

## Supplementary Figures

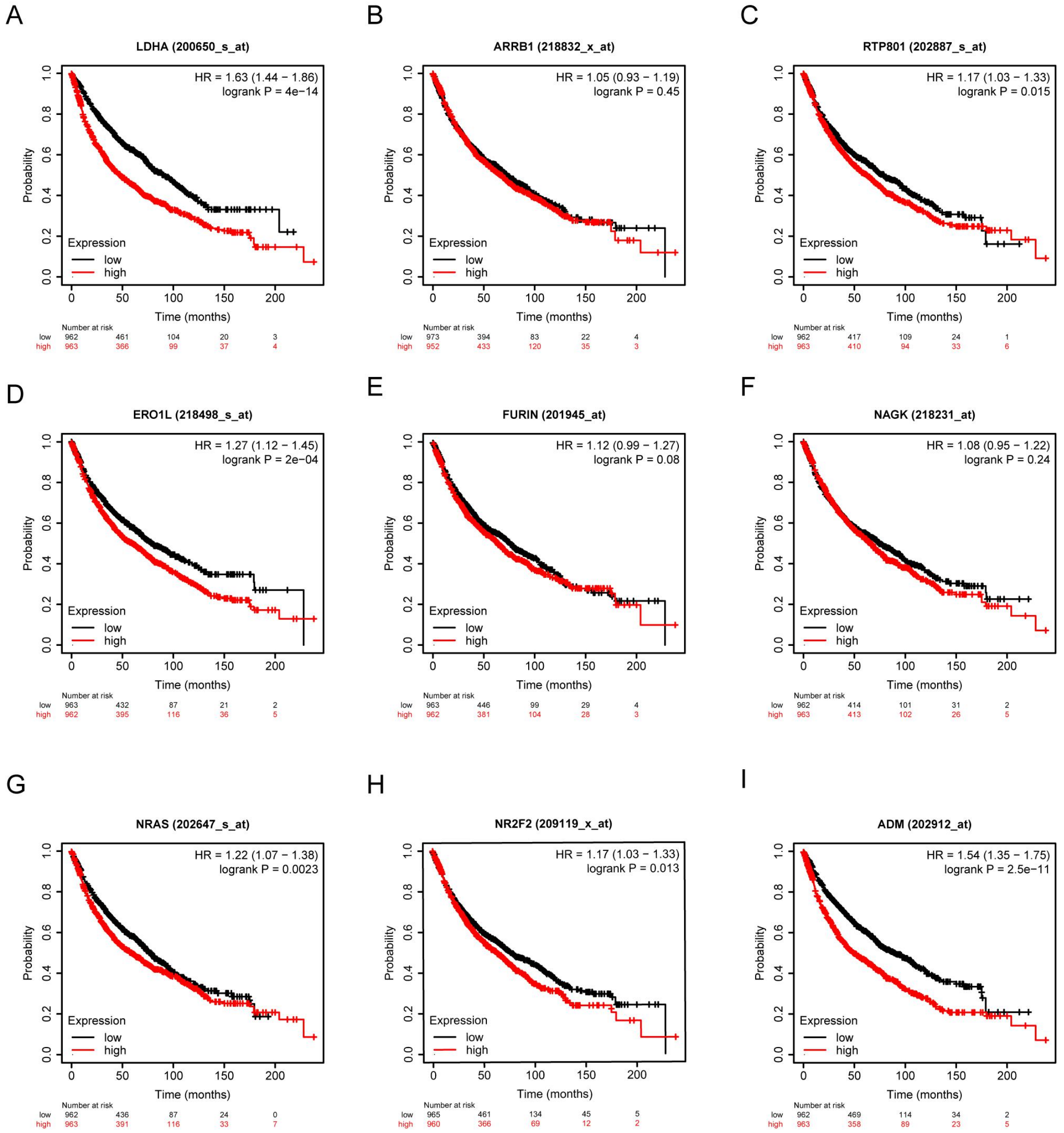


**Figure S1. Kaplan–Meier analysis of 9 genes involved in model construction (LDHA, ARRB1, DDIT4, EROIL, FURIN, NAGK, NRAS, NR2F2, and ADM).**

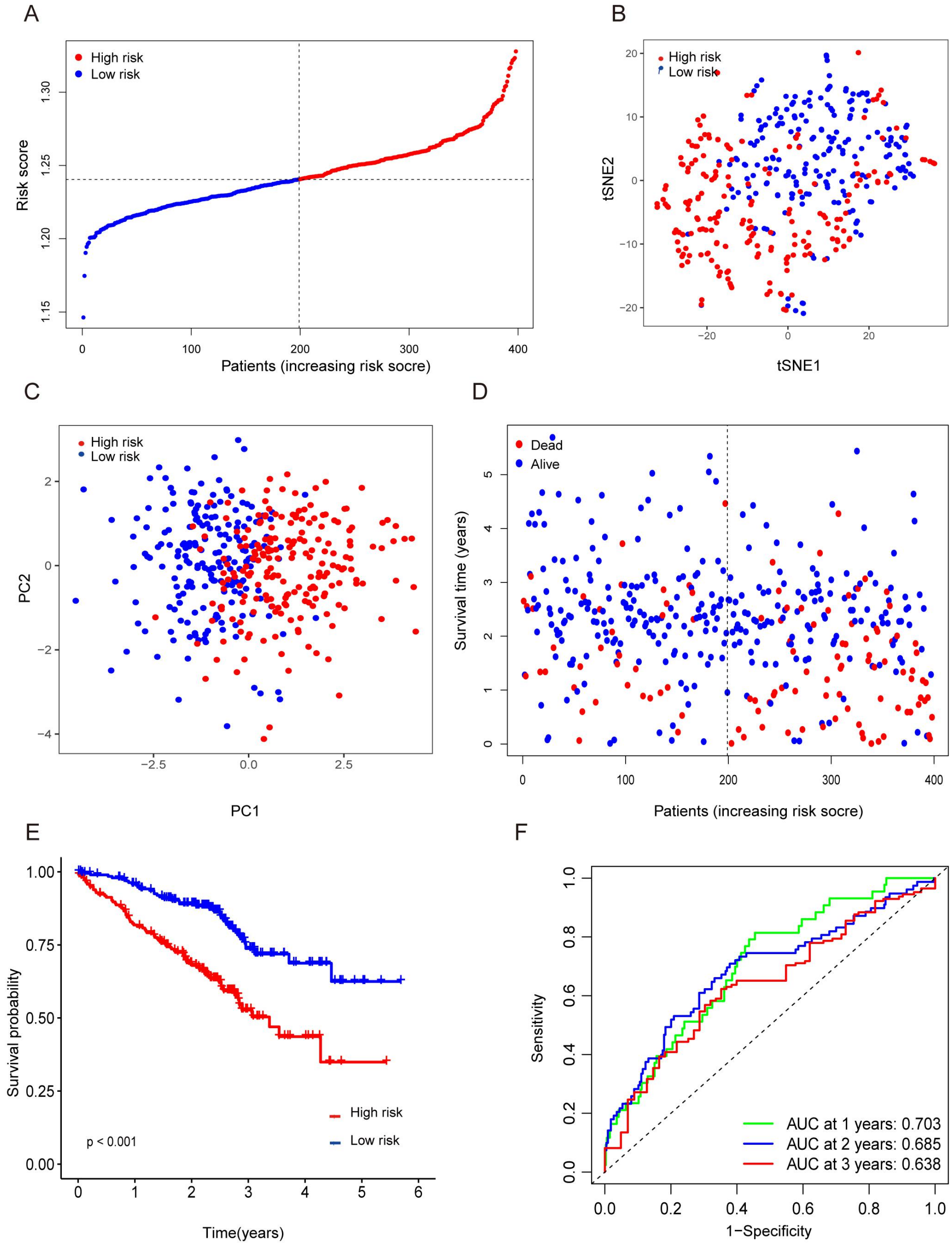


**Figure S2. Validation of the nine-genes signature in the GSE72094 cohort. (A)** The distributions of the risk score. **(B, C)** t-SNE and PCA analysis showed significant differences between groups of patients. **(D, E)** The distributions of OS status and OS of patients between high-risk and low-risk groups, patients in the high-risk group had higher score values and mortality. **(F)** Time-independent ROC analysis of the risk score for prediction of the OS, the area under the curve for 1, 2, and 3 years reached 0.703, 0.685, and 0.638, respectively.

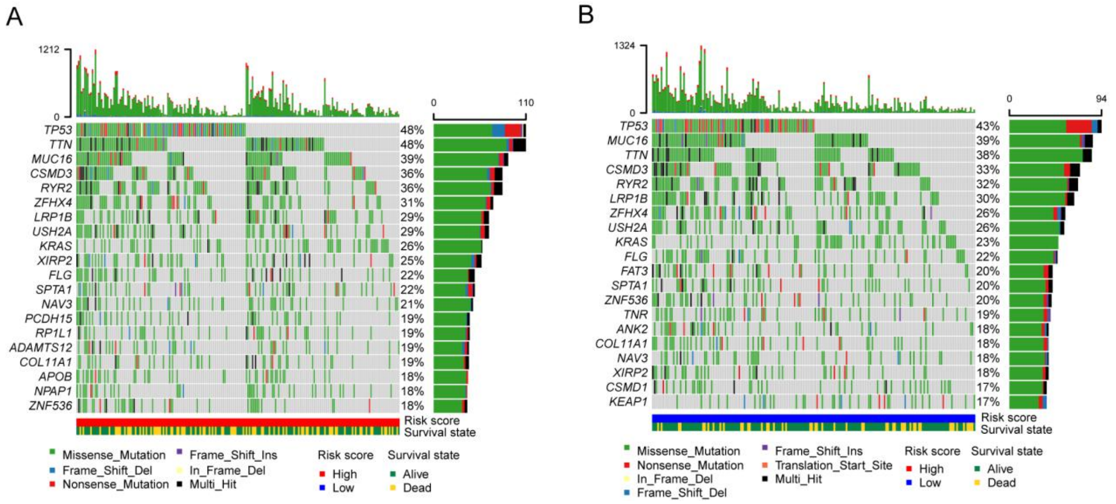


**Figure S3. Somatic mutation in the risk score stratified groups. (A, B)** Oncoplots of the mutated genes in the (**A**) high-risk and (**B**) low-risk subgroups of the TCGA cohort.

## Supplementary Tables

Table S1. The clinical characteristics of the TCGA, GSE31210 and GSE72094 cohorts.

| Clinical characteristic | | TCGA cohort | GSE31210 cohort | GSE72094 cohort |
|---|---|---|---|---|
| Age | <65 | 215 | 164 | 107 |
| | ≥65 | 254 | 62 | 291 |
| Gender | Male | 213 | 105 | 176 |
| | Female | 256 | 121 | 222 |
| Stage | Ⅰ - Ⅱ | 367 | 226 | 321 |

| | | | | |
|---|---|---|---|---|
| | Ⅲ-Ⅳ | 102 | 0 | 72 |
| | Unknown | | | 5 |

Table S2. Summary of hypoxia-related genes.

| Hypoxia-related genes | | | | |
|---|---|---|---|---|
| ACKR3 | CSRP2 | HDLBP | NDRG1 | NDRG1 |
| ADM | CXCR4 | HEXA | NDST1 | NDST1 |
| ADORA2B | DCN | HK1 | NDST2 | NDST2 |
| AK4 | DDIT3 | HK2 | NEDD4L | NEDD4L |
| AKAP12 | DDIT4 | HMOX1 | NFIL3 | NFIL3 |
| ALDOA | DPYSL4 | HOXB9 | NOCT | NOCT |
| ALDOB | DTNA | HS3ST1 | NR3C1 | NR3C1 |
| ALDOC | DUSP1 | HSPA5 | P4HA1 | P4HA1 |
| AMPD3 | EDN2 | IDS | P4HA2 | P4HA2 |
| ANGPTL4 | EFNA1 | IER3 | PAM | PAM |
| ANKZF1 | EFNA3 | IGFBP1 | PCK1 | PCK1 |
| ANXA2 | EGFR | IGFBP3 | PDGFB | PDGFB |
| ATF3 | ENO1 | IL6 | PDK1 | PDK1 |
| ATP7A | ENO2 | ILVBL | PDK3 | PDK3 |
| B3GALT6 | ENO3 | INHA | PFKFB3 | PFKFB3 |
| B4GALNT2 | ERO1A | IRS2 | PFKL | PFKL |
| BCAN | ERRFI1 | ISG20 | PFKP | PFKP |
| BCL2 | ETS1 | JMJD6 | PGAM2 | PGAM2 |
| BGN | EXT1 | JUN | PGF | PGF |
| BHLHE40 | F3 | KDELR3 | PGK1 | PGK1 |
| BNIP3L | FAM162A | KDM3A | PGM1 | PGM1 |
| BRS3 | FBP1 | KIF5A | PGM2 | PGM2 |
| BTG1 | FOS | KLF6 | PHKG1 | PHKG1 |
| CA12 | FOSL2 | KLF7 | PIM1 | PIM1 |
| CASP6 | FOXO3 | KLHL24 | PKLR | PKLR |
| CAV1 | GAA | LALBA | PKP1 | PKP1 |
| CAVIN1 | GALK1 | LARGE1 | PLAC8 | PLAC8 |
| CAVIN3 | GAPDH | LDHA | PLAUR | PLAUR |

| | | | | |
|---|---|---|---|---|
| CCN1 | GAPDHS | LDHC | PLIN2 | PLIN2 |
| CCN2 | GBE1 | LOX | PNRC1 | PNRC1 |
| CCN5 | GCK | LXN | PPARGC1A | PPARGC1A |
| CCNG2 | GCNT2 | MAFF | PPFIA4 | PPFIA4 |
| CDKN1A | GLRX | MAP3K1 | PPP1R15A | PPP1R15A |
| CDKN1B | GPC1 | MIF | PPP1R3C | PPP1R3C |
| CDKN1C | GPC3 | MT1E | PRDX5 | PRDX5 |
| CHST2 | GPC4 | MT2A | PRKCA | PRKCA |
| CHST3 | GPI | MXI1 | PYGM | PYGM |
| CITED2 | GRHPR | MYH9 | RBPJ | RBPJ |
| COL5A1 | GYS1 | NAGK | RORA | RORA |
| CP | HAS1 | NCAN | RRAGD | RRAGD |

Table S3. Summary of immune-related genes.

| Immune-related genes | | | | |
|---|---|---|---|---|
| AZGP1 | PRTN3 | IGHD4-11 | FGF4 | NR1I3 |
| B2M | MAPK1 | IGHD4-17 | FGF5 | NR2C1 |
| CALR | PML | IGHD4-23 | FGF6 | NR2C2 |
| CANX | AEN | IGHD4-4 | FGF7 | NR2E1 |
| CD1A | CYBB | IGHD5-12 | FGF8 | NR2E3 |
| CD1B | BPIFA2 | IGHD5-18 | FGF9 | NR2F1 |
| CD1C | ISG20 | IGHD5-24 | VEGFD | NR2F2 |
| CD1D | BCL3 | IGHD5-5 | FIGNL2 | NR2F6 |
| CD1E | ISG20L2 | IGHD6-13 | FLT3LG | NR3C1 |
| CD4 | NOX5 | IGHD6-19 | FSHB | NR3C2 |
| CD8A | NOX3 | IGHD6-25 | GAL | NR4A1 |
| CD8B | DUOX2 | IGHD6-6 | GALP | NR4A2 |
| CD74 | TLR3 | IGHD7-27 | GAST | NR4A3 |
| CREB1 | TFRC | IGHE | GCG | NR5A1 |
| CTSB | IFIH1 | IGHG1 | GDF1 | NR5A2 |
| CTSE | LRP1 | IGHG2 | GDF10 | NR6A1 |
| CTSL | TRIM5 | IGHG3 | GDF11 | NRP1 |
| CTSS | IDO1 | IGHG4 | GDF2 | NRP2 |
| FCER1G | GDF15 | IGHJ1 | GDF3 | OGFR |
| FCGRT | NEDD4 | IGHJ2 | GDF5 | OPRD1 |
| PDIA3 | ADIPOQ | IGHJ3 | GDF6 | OPRK1 |
| HFE | STAT3 | IGHJ4 | GDF7 | OPRL1 |
| HLA-A | STAT1 | IGHJ5 | GDF9 | OPRM1 |
| HLA-B | IFNL2 | IGHJ6 | GDNF | OSMR |
| HLA-C | SOCS3 | IGHM | GH1 | OXTR |
| HLA-DMA | SEMG1 | IGHV1-18 | GH2 | PGR |

| | | | | |
|---|---|---|---|---|
| HLA-DMB | TNFSF10 | IGHV1-2 | GHRH | PGRMC2 |
| HLA-DOA | CCL20 | IGHV1-24 | GHRL | PPARA |
| HLA-DOB | SOCS1 | IGHV1-3 | GIP | PPARD |
| HLA-DPA1 | RNASEL | IGHV1-45 | GKN1 | PRLHR |
| HLA-DPB1 | IRF1 | IGHV1-46 | GMFB | PRLR |
| HLA-DQA1 | IL15 | IGHV1-58 | GMFG | PTGER1 |
| HLA-DQA2 | APOBEC3F | IGHV1-69 | GNRH1 | PTGER2 |
| HLA-DQB1 | PLAAT4 | IGHV1-8 | GNRH2 | PTGER3 |
| HLA-DRA | CHIT1 | IGHV1-38-4 | GPHA2 | PTGER4 |
| HLA-DRB1 | CD40 | IGHV1-69-2 | GPHB5 | PTGFR |
| HLA-DRB3 | TLR7 | IGHV2-26 | GPI | PTH1R |
| HLA-DRB4 | PPIA | IGHV2-5 | GREM1 | PTH2R |
| HLA-DRB5 | ZYX | IGHV2-70 | GREM2 | RARA |
| HLA-E | NLRX1 | IGHV3-11 | GRP | RARB |
| HLA-F | PGC | IGHV3-13 | GUCA2A | RARG |
| HLA-G | VEGFA | IGHV3-15 | HBEGF | RORA |
| HLA-H | IKBKE | IGHV3-16 | HDGF | RORB |
| MR1 | ISG15 | IGHV3-20 | HDGFL3 | RORC |
| HSPA1A | DHX58 | IGHV3-21 | IAPP | RXFP1 |
| HSPA1B | TNFAIP3 | IGHV3-23 | IFNE | RXFP2 |
| HSPA1L | TFR2 | IGHV3-30 | IFNK | RXRA |
| HSPA2 | FCN2 | IGHV3-30-3 | IFNW1 | RXRB |
| HSPA4 | MUC4 | IGHV3-30-5 | IGF1 | RXRG |
| HSPA5 | F2R | IGHV3-33 | IGF2 | S1PR1 |
| HSPA6 | ELN | IGHV3-35 | IL11 | S1PR2 |
| HSPA8 | IL27 | IGHV3-38 | IL12A | SCTR |
| HSP90AA1 | MAPT | IGHV3-43 | IL16 | SDC1 |
| HSP90AB1 | LYZ | IGHV3-48 | IL17B | SDC2 |
| ICAM1 | CCL5 | IGHV3-49 | IL17C | SDC3 |
| IFNA1 | LEP | IGHV3-53 | IL17D | SDC4 |
| IFNA2 | CYLD | IGHV3-64 | IL17F | SORT1 |
| IFNA4 | KLKB1 | IGHV3-66 | IL19 | SSTR1 |
| IFNA5 | CST4 | IGHV3-7 | IL1F10 | SSTR2 |
| IFNA6 | CSRP1 | IGHV3-72 | IL36RN | SSTR5 |
| IFNA7 | MAPK14 | IGHV3-73 | IL36A | ST2 |
| IFNA8 | JUN | IGHV3-74 | IL37 | TACR1 |
| IFNA10 | ITGAV | IGHV3-9 | IL36B | TEK |
| IFNA13 | IRF5 | IGHV3-38-3 | IL36G | TGFBR1 |
| IFNA14 | CCR6 | IGHV3-69-1 | IL1RN | TGFBR2 |
| IFNA16 | IL12B | IGHV4-28 | IL20 | TGFBR3 |
| IFNA17 | TLR8 | IGHV4-30-1 | IL21 | THRA |
| IFNA21 | GNLY | IGHV4-30-2 | IL23A | THRB |
| IFNG | CD81 | IGHV4-30-4 | IL24 | TIE1 |
| KIR2DL1 | EIF2AK2 | IGHV4-31 | IL25 | TNFRSF10C |

| | | | | |
|---|---|---|---|---|
| KIR2DL2 | APOM | IGHV4-34 | IL26 | TNFRSF10D |
| KIR2DL3 | CACYBP | IGHV4-39 | IFNL3 | TNFRSF11A |
| KIR2DL4 | NOD1 | IGHV4-4 | IL3 | TNFRSF12A |
| KIR2DS1 | MAPK8 | IGHV4-59 | IL31 | TNFRSF13B |
| KIR2DS3 | MAPK3 | IGHV4-61 | IL32 | TNFRSF13C |
| KIR2DS4 | BST2 | IGHV4-38-2 | IL33 | TNFRSF14 |
| KIR2DS5 | BPHL | IGHV5-51 | IL34 | TNFRSF17 |
| KIR3DL1 | PLA2G2A | IGHV5-10-1 | IL5 | TNFRSF18 |
| KIR3DL2 | GRN | IGHV6-1 | IL6ST | TNFRSF19 |
| KLRC1 | NEWENTRY | IGHV7-4-1 | IL7 | TNFRSF1A |
| KLRC2 | PDGFRA | IGHV7-81 | IL9 | TNFRSF1B |
| KLRC3 | GNAI1 | IGK | INHA | TNFRSF21 |
| KLRD1 | WNT5A | IGKC | INHBA | TNFRSF25 |
| LTA | FURIN | IGKDEL | INHBB | TNFRSF4 |
| CIITA | ADAR | IGKJ | INHBC | TNFRSF6B |
| MICA | TYK2 | IGKJ1 | INHBE | TNFRSF8 |
| MICB | NOS2 | IGKJ2 | INS | TNFRSF9 |
| NFYA | TRAF3 | IGKJ3 | INS-IGF2 | TRHR |
| NFYB | TPT1 | IGKJ4 | INSL3 | TSHR |
| NFYC | TPM2 | IGKJ5 | INSL4 | TUBB3 |
| LGMN | NEO1 | IGKV@ | INSL5 | VIPR1 |
| PSMB8 | AHNAK | IGKV1-12 | INSL6 | VIPR2 |
| PSMC1 | TLR1 | IGKV1-13 | JAG1 | PTPN11 |
| PSMC2 | TK2 | IGKV1-16 | JAG2 | ICAM2 |
| PSMC3 | PRDX2 | IGKV1-17 | FGF7P6 | ITGAL |
| PSMC4 | MX2 | IGKV1-27 | FGF7P3 | ITGB2 |
| PSMC5 | FGF2 | IGKV1-33 | KITLG | PAK1 |
| PSMC6 | FGA | IGKV1-37 | KL | NCR2 |
| PSMD1 | TCF7L2 | IGKV1-39 | LACRT | TYROBP |
| PSMD2 | F2RL1 | IGKV1-5 | LEFTY1 | LCK |
| PSMD3 | TKFC | IGKV1-6 | LEFTY2 | FCGR3A |
| PSMD4 | MSR1 | IGKV1-8 | LHB | FCGR3B |
| PSMD5 | NFKBIZ | IGKV1-9 | LIF | NCR1 |
| PSMD7 | LMBR1 | IGKV1D-12 | LRSAM1 | NCR3 |
| PSMD8 | EPPIN | IGKV1D-13 | LTB | CD247 |
| PSMD10 | SRC | IGKV1D-16 | LTBP2 | ZAP70 |
| PSMD11 | MPO | IGKV1D-17 | LTBP3 | LCP2 |
| PSMD13 | ELAVL1 | IGKV1D-33 | LTBP4 | LAT |
| PSME1 | ROBO3 | IGKV1D-37 | MDK | PLCG1 |
| PSME2 | SP1 | IGKV1D-39 | MIA | SH3BP2 |
| RELB | SOD1 | IGKV1D-42 | MLN | FYN |
| RFX5 | PDF | IGKV1D-43 | MSTN | SHC2 |
| RFXAP | DLL4 | IGKV1D-8 | NAMPT | SHC4 |
| SLC10A2 | ECD | IGKV2-24 | NDP | SHC3 |

| | | | | |
|---|---|---|---|---|
| TAP1 | SLC11A1 | IGKV2-28 | NENF | SHC1 |
| TAP2 | DMBT1 | IGKV2-30 | NGF | GRB2 |
| TAPBP | STING1 | IGKV2-40 | NMB | SOS1 |
| THBS1 | SKIV2L | IGKV2D-24 | NODAL | SOS2 |
| SEM1 | SEMG2 | IGKV2D-28 | CCN3 | ARAF |
| KLRC4 | DES | IGKV2D-29 | NPFF | BRAF |
| AP3B1 | DCK | IGKV2D-30 | NPPA | RAF1 |
| RFXANK | DAXX | IGKV2D-40 | NPPB | HCST |
| PSMD6 | TNFRSF10A | IGKV3-11 | NPPC | CD48 |
| PSME3 | TNFRSF10B | IGKV3-15 | NPY | CD244 |
| PSMD14 | EED | IGKV3-20 | NRG1 | PRKCA |
| CLEC4M | CCL4 | IGKV3-7 | NRG2 | PRKCG |
| IFI30 | LIMS1 | IGKV3D-11 | NRG3 | SH2D1B |
| PROCR | LALBA | IGKV3D-15 | NRG4 | SH2D1A |
| ADRM1 | APOBEC3H | IGKV3D-20 | NRTN | FAS |
| ECPAS | TMPRSS6 | IGKV3D-7 | NTF3 | GZMB |
| TRPC4AP | SPINK5 | IGKV4-1 | NTF4 | PRF1 |
| CD209 | MARCO | IGKV5-2 | NTS | CASP3 |
| UBXN1 | BECN1 | IGKV6-21 | NUDT6 | BID |
| ERAP1 | TNFSF11 | IGKV6D-21 | OGN | CD3D |
| TAPBPL | KNG1 | IGKV6D-41 | OSGIN1 | CD3E |
| KIR2DL5A | CSK | IGL | OSM | CD3G |
| ERAP2 | KLRK1 | IGLC1 | OSTN | PTPRC |
| ULBP3 | KCNH2 | IGLC2 | OXT | ITK |
| ULBP2 | JUND | IGLC3 | ENDOU | TEC |
| ULBP1 | JAK1 | IGLC6 | PDGFA | NCK1 |
| KIR3DL3 | CLDN4 | IGLC7 | PDGFB | NCK2 |
| RAET1E | CCL28 | IGLJ | PDGFC | GRAP2 |
| RAET1L | RNASE3 | IGLJ1 | PDGFD | PAK2 |
| UBR1 | RN7SL1 | IGLJ2 | PDGFRL | PAK3 |
| RAET1G | IRF7 | IGLJ3 | PGF | PAK4 |
| PDIA2 | IREB2 | IGLJ4 | PMCH | PAK6 |
| HAMP | ILK | IGLJ5 | PNOC | PAK5 |
| PI3 | IL18 | IGLJ6 | POMC | RHOA |
| CAMP | IL17A | IGLJ7 | PPBPP2 | CDC42 |
| DEFB4A | LTB4R | IGLV@ | PPY | CD28 |
| PPBP | APOBEC3A | IGLV1-36 | PRL | ICOS |
| REG3G | MASP2 | IGLV1-40 | PRLH | MAP3K8 |
| CXCL14 | TRIM27 | IGLV1-44 | PROK1 | MAP3K14 |
| CXCL16 | RELA | IGLV1-47 | PSPN | CTLA4 |
| SLPI | IL7R | IGLV1-50 | PTH | CBLC |
| CXCL8 | IL1A | IGLV1-51 | PTH2 | CBL |
| CXCL10 | PTX3 | IGLV10-54 | PTHLH | CBLB |
| CXCL9 | IFNAR2 | IGLV11-55 | PTN | CDK4 |

| | | | | |
|---|---|---|---|---|
| CXCL5 | IFN1@ | IGLV2-11 | PYY | RASGRP1 |
| CXCL11 | SYTL1 | IGLV2-14 | QRFP | PDK1 |
| CXCL6 | APOBEC3C | IGLV2-18 | RABEP1 | PRKCQ |
| CXCL1 | DDX17 | IGLV2-23 | RABEP2 | TRAC |
| CXCL12 | PTGS2 | IGLV2-33 | REG1A | TRAJ1 |
| CXCL13 | HTR1A | IGLV2-8 | RETN | TRAJ2 |
| CXCL2 | SEPTIN7 | IGLV3-1 | RETNLB | TRAJ3 |
| PF4 | CD40LG | IGLV3-10 | RLN1 | TRAJ4 |
| XCL1 | CD14 | IGLV3-12 | RLN2 | TRAJ5 |
| CXCL3 | MASP1 | IGLV3-16 | RLN3 | TRAJ6 |
| DEFB103B | PROC | IGLV3-19 | SCG2 | TRAJ7 |
| CCL13 | MAP2K2 | IGLV3-21 | SCGB3A1 | TRAJ8 |
| CCL1 | MAP2K1 | IGLV3-22 | SCT | TRAJ9 |
| DEFB1 | HRG | IGLV3-25 | AIMP1 | TRAJ10 |
| CCL8 | NDRG1 | IGLV3-27 | SECTM1 | TRAJ11 |
| ELANE | IRF9 | IGLV3-32 | SLURP1 | TRAJ12 |
| DEFB103A | TRIM22 | IGLV3-9 | SPP1 | TRAJ13 |
| DEFA3 | LANCL1 | IGLV4-3 | SST | TRAJ14 |
| DEFA1 | PPP4C | IGLV4-60 | STC1 | TRAJ15 |
| TMSB10 | HMOX1 | IGLV4-69 | STC2 | TRAJ16 |
| DEFA6 | HMGB1 | IGLV5-37 | TAC1 | TRAJ17 |
| DEFA5 | RNASE7 | IGLV5-39 | TDGF1 | TRAJ18 |
| DEFA4 | ABCC4 | IGLV5-45 | TDGF1P3 | TRAJ19 |
| LCN2 | HGF | IGLV5-48 | TG | TRAJ20 |
| LCN1 | HDAC1 | IGLV5-52 | TGFA | TRAJ21 |
| COLEC10 | IFNLR1 | IGLV6-57 | TGFB2 | TRAJ22 |
| BPI | PLSCR1 | IGLV7-43 | TGFB3 | TRAJ23 |
| S100A9 | BACH2 | IGLV7-46 | THPO | TRAJ24 |
| S100A8 | TANK | IGLV8-61 | TNFRSF11B | TRAJ25 |
| DCD | PIK3CG | IGLV9-49 | TNFSF12 | TRAJ26 |
| LCN6 | ARRB1 | C3 | TNFSF13 | TRAJ27 |
| S100A12 | RSAD2 | C5 | TNFSF13B | TRAJ28 |
| HTN3 | STAB2 | CCL3P1 | TNFSF14 | TRAJ29 |
| LCN8 | TBK1 | CKLF | TNFSF15 | TRAJ30 |
| DEFA1B | PDYN | CMA1 | TNFSF18 | TRAJ31 |
| CCR10 | PDGFRB | CX3CL1 | TNFSF8 | TRAJ32 |
| CELA1 | PDCD1 | CXCL17 | TNFSF9 | TRAJ33 |
| DEFB106A | PCSK2 | CCN1 | TOR2A | TRAJ34 |
| PENK | PCSK1 | EDN1 | TRH | TRAJ35 |
| BPIFC | ARG2 | EDN2 | TSHB | TRAJ36 |
| MMP12 | AQP9 | EDN3 | TSLP | TRAJ37 |
| BPIFB6 | FASLG | FGF10 | TXLNA | TRAJ38 |
| LEAP2 | APOH | LECT2 | UCN | TRAJ39 |
| SFTPD | BIRC5 | PPBPP1 | UCN2 | TRAJ40 |

| | | | | |
|---|---|---|---|---|
| LCN9 | ANXA6 | PROK2 | UCN3 | TRAJ41 |
| BPIFB2 | IL22 | SAA1 | UTS2 | TRAJ42 |
| PTGDS | VTN | SAA2 | UTS2B | TRAJ43 |
| TMSB4X | VIM | SBDS | VEGFB | TRAJ44 |
| PGLYRP1 | VCAM1 | SEMA3A | VEGFC | TRAJ45 |
| ZC3HAV1 | PRDX1 | SEMA3B | VGF | TRAJ46 |
| TMSB15A | GFAP | SEMA3C | VIP | TRAJ47 |
| S100B | GBP2 | SEMA3D | ACVR1B | TRAJ48 |
| S100A13 | ALB | SEMA3E | ACVR1C | TRAJ49 |
| S100A6 | SLC29A3 | SEMA3F | ACVR2A | TRAJ50 |
| DEFB119 | OAS1 | SEMA3G | ACVR2B | TRAJ52 |
| DEFB107A | AGER | SEMA4A | ACVRL1 | TRAJ53 |
| DEFB105A | UNC93B1 | SEMA4B | ADCYAP1R1 | TRAJ54 |
| SERPIND1 | TNFSF4 | SEMA4C | ADIPOR1 | TRAJ56 |
| DEFB129 | NOS1 | SEMA4D | ADIPOR2 | TRAJ57 |
| DEFB127 | ACTG1 | SEMA4F | ADRB1 | TRAJ58 |
| S100P | ACTA1 | SEMA4G | ADRB2 | TRAJ59 |
| S100A7 | ACO1 | SEMA5A | AGTR1 | TRAJ61 |
| DEFB104A | SERPINA3 | SEMA5B | AGTR2 | TRAV1-1 |
| DEFB126 | CXCR1 | SEMA6A | AMHR2 | TRAV1-2 |
| DEFB106B | CCL15 | SEMA6B | ANGPT1 | TRAV2 |
| DEFB104B | CCL14 | SEMA6C | ANGPT4 | TRAV3 |
| DEFB107B | CCL16 | SEMA6D | ANGPTL1 | TRAV4 |
| PGLYRP3 | CCL19 | SEMA7A | ANGPTL2 | TRAV5 |
| PGLYRP2 | CCL18 | SLIT1 | ANGPTL3 | TRAV7 |
| S100A10 | CCL17 | SLIT2 | ANGPTL4 | TRAV8-1 |
| S100A2 | CCL26 | TNC | ANGPTL6 | TRAV8-2 |
| DEFB125 | CCL22 | TYMP | APLNR | TRAV8-3 |
| DEFB123 | CCR3 | C5AR1 | AR | TRAV8-4 |
| DEFB105B | CCL4L1 | CCR9 | AVPR1A | TRAV8-6 |
| DEFB132 | ACKR2 | CCRL2 | AVPR1B | TRAV8-7 |
| BPIFB3 | CCR7 | CMKLR1 | AVPR2 | TRAV9-1 |
| LCN12 | CCL27 | CX3CR1 | BMPR1A | TRAV9-2 |
| PGLYRP4 | CCR8 | CXCR3 | BMPR1B | TRAV10 |
| S100A11 | ACKR4 | CXCR5 | BMPR2 | TRAV12-1 |
| S100A5 | CCL2 | ACKR3 | BRD8 | TRAV12-2 |
| S100A3 | CCL21 | CYSLTR1 | C3AR1 | TRAV12-3 |
| S100A1 | CCL7 | CYSLTR2 | CALCR | TRAV13-1 |
| DEFB128 | CCL3 | ACKR1 | CALCRL | TRAV13-2 |
| DEFB108B | CCL11 | EDNRA | CNTFR | TRAV14DV4 |
| HTN1 | CCR5 | EDNRB | CRHR1 | TRAV16 |
| LMBR1L | CCL23 | FPR1 | CRHR2 | TRAV17 |
| S100A7A | CCL25 | FPR2 | CRIM1 | TRAV18 |

| | | | | |
|---|---|---|---|---|
| DEFB118 | CCL3L3 | GPR17 | CRLF1 | TRAV19 |
| COLEC12 | CCL4L2 | GPR32 | CRLF2 | TRAV20 |
| TMSB4Y | CCL3L1 | GPR33 | CRLF3 | TRAV21 |
| DEFB131A | CCR1 | PTGDR2 | CSF1R | TRAV22 |
| DEFB134 | CCL24 | C5AR2 | CSF2RA | TRAV23DV6 |
| DEFB130A | XCL2 | CXCR2 | CSF2RB | TRAV24 |
| DEFB124 | CXCR4 | LTB4R2 | CSF3R | TRAV25 |
| DEFB121 | CXCR6 | PLAUR | EGFR | TRAV26-1 |
| DEFB116 | CCR4 | PLXNA1 | ENG | TRAV26-2 |
| DEFB115 | TAFA5 | PLXNA2 | EPOR | TRAV27 |
| DEFB114 | TAFA3 | PLXNA3 | ESR1 | TRAV29DV5 |
| DEFB113 | TAFA4 | PLXNA4 | ESR2 | TRAV30 |
| DEFB112 | TAFA1 | PLXNB1 | ESRRA | TRAV34 |
| DEFB110 | TAFA2 | PLXNB2 | ESRRB | TRAV35 |
| TMSB15B | CCL15-CCL14 | PLXNB3 | ESRRG | TRAV36DV7 |
| DEFB133 | PTK2B | PLXNC1 | FGFR1 | TRAV38-1 |
| S100Z | IL4 | PLXND1 | FGFR2 | TRAV38-2DV8 |
| MAVS | CDH1 | PTAFR | FGFR3 | TRAV39 |
| TMSB4XP8 | LTBP1 | ROBO1 | FGFR4 | TRAV40 |
| S100A14 | IL13 | ROBO2 | FGFRL1 | TRAV41 |
| LCN10 | IL10 | RXFP3 | FLT1 | TRBC1 |
| S100A16 | IL2 | XCR1 | FLT3 | TRBC2 |
| DEFB136 | PPARG | ADM | FLT4 | TRBD1 |
| DEFB135 | FGR | ADM2 | FSHR | TRBD2 |
| DEFB117 | MIF | AGRP | GALR2 | TRBJ1-1 |
| ZC3HAV1L | CRP | AGT | GALR3 | TRBJ1-2 |
| S100A7L2 | JAK2 | AMBN | GCGR | TRBJ1-3 |
| MBL3P | PTK2 | AMELX | GHR | TRBJ1-4 |
| DEFB4B | PTGDR | AMH | GHRHR | TRBJ1-5 |
| BPIFB4 | CD86 | ANGPTL5 | GHSR | TRBJ1-6 |
| IFNAR1 | HCK | ANGPTL7 | GIPR | TRBJ2-1 |
| AZU1 | VDR | APLN | GLP1R | TRBJ2-2 |
| DEFB131B | OLR1 | AREG | GLP2R | TRBJ2-3 |
| DEFA1A3 | GRK2 | MANF | GNRHR | TRBJ2-4 |
| LCN1P1 | TXK | CDNF | GPER1 | TRBJ2-5 |
| S100G | RNASE2 | ARTN | HNF4A | TRBJ2-6 |
| DEFA7P | CD79A | AVP | HNF4G | TRBJ2-7 |
| DEFB130B | CD79B | BDNF | HTR3A | TRBV2 |
| DEFB108F | LYN | BMP1 | HTR3B | TRBV3-1 |
| DEFB131C | SYK | BMP10 | HTR3C | TRBV4-1 |
| TCHHL1 | BTK | BMP15 | HTR3D | TRBV4-2 |
| TINAGL1 | BLNK | BMP2 | HTR3E | TRBV4-3 |

| | | | | |
|---|---|---|---|---|
| IFNGR1 | VAV3 | BMP3 | IFNGR2 | TRBV5-1 |
| SLC22A17 | VAV1 | BMP4 | IGF1R | TRBV5-4 |
| WFIKKN1 | VAV2 | BMP5 | IGF2R | TRBV5-5 |
| WFDC2 | RAC1 | BMP6 | IL10RA | TRBV5-6 |
| IL6 | RAC2 | BMP7 | IL10RB | TRBV5-7 |
| UMODL1 | RAC3 | BMP8A | IL11RA | TRBV5-8 |
| TGFB1 | PPP3CA | BMP8B | IL12RB1 | TRBV6-1 |
| PF4V1 | PPP3CB | BTC | IL12RB2 | TRBV6-2 |
| MMP9 | PPP3CC | MYDGF | IL13RA1 | TRBV6-3 |
| ANOS1 | CHP1 | CALCA | IL13RA2 | TRBV6-4 |
| TLR4 | PPP3R1 | CALCB | IL15RA | TRBV6-5 |
| SPAG11B | PPP3R2 | CAT | IL2RB | TRBV6-6 |
| A2M | CHP2 | CCK | IL17RA | TRBV6-7 |
| NFKB1 | NFAT5 | CD320 | IL17RB | TRBV6-8 |
| APOBEC3G | NFATC1 | CD70 | IL17RC | TRBV6-9 |
| FABP6 | NFATC2 | ADA2 | IL17RD | TRBV7-2 |
| NOD2 | NFATC3 | CER1 | IL17RE | TRBV7-3 |
| MBL2 | NFATC4 | CGA | IL18R1 | TRBV7-4 |
| SFTPA1 | HRAS | CGB3 | IL18RAP | TRBV7-6 |
| RBP1 | KRAS | CGB1 | IL1R1 | TRBV7-7 |
| TLR2 | NRAS | CGB2 | IL1R2 | TRBV7-8 |
| SLC40A1 | FOS | CGB5 | IL1RAP | TRBV7-9 |
| PLAU | CARD11 | CGB7 | IL1RL1 | TRBV9 |
| IL1B | BCL10 | CGB8 | IL1RL2 | TRBV10-1 |
| PAEP | MALT1 | CHGA | IL20RA | TRBV10-2 |
| HJV | CHUK | CHGB | IL20RB | TRBV10-3 |
| MUC5AC | IKBKB | CLCF1 | IL21R | TRBV11-1 |
| OBP2A | IKBKG | CLEC11A | IL22RA1 | TRBV11-2 |
| PLTP | NFKBIA | CMTM1 | IL22RA2 | TRBV11-3 |
| MX1 | NFKBIB | CMTM2 | IL23R | TRBV12-3 |
| DDX58 | NFKBIE | CMTM3 | IL27RA | TRBV12-4 |
| IFNL1 | CD19 | CMTM4 | IL2RA | TRBV12-5 |
| IRF3 | CR2 | CMTM5 | IL2RG | TRBV13 |
| SFTPA2 | PIK3R5 | CMTM6 | IL31RA | TRBV14 |
| LPA | PIK3R1 | CMTM7 | IL3RA | TRBV15 |
| LBP | PIK3R2 | CMTM8 | IL4R | TRBV16 |
| RBP4 | PIK3R3 | CNTF | IL5RA | TRBV17 |
| NOX4 | PIK3CA | CORT | IL6R | TRBV18 |
| LTF | PIK3CB | CRH | IL9R | TRBV19 |
| IFNB1 | PIK3CD | CSF1 | INSR | TRBV20-1 |
| RBP5 | AKT3 | CSF2 | KDR | TRBV24-1 |
| FABP7 | AKT1 | CSF3 | LEPR | TRBV25-1 |
| FABP5 | AKT2 | CSH1 | LGR4 | TRBV27 |
| FABP3 | GSK3B | CSH2 | LGR5 | TRBV28 |

| | | | | |
|---|---|---|---|---|
| FABP2 | INPP5D | CSHL1 | LGR6 | TRBV29-1 |
| FABP4 | CD22 | CSPG5 | LHCGR | TRBV30 |
| R3HDML | CD72 | CTF1 | LIFR | TRDC |
| BPIFA3 | PTPN6 | CCN2 | LTBR | TRDD1 |
| BPIFB1 | LILRB3 | DKK1 | MC1R | TRDD2 |
| OASL | FCGR2B | EBI3 | MC2R | TRDD3 |
| CRABP2 | RASGRP3 | EGF | MC3R | TRDJ1 |
| CRABP1 | PLCG2 | EPGN | MC4R | TRDJ2 |
| RBP7 | PRKCB | EPO | MCHR1 | TRDJ3 |
| DUOX1 | IFITM1 | EREG | MCHR2 | TRDJ4 |
| OBP2B | IGH | ESM1 | MET | TRDV1 |
| RBP2 | IGHA1 | FAM3B | MLNR | TRDV2 |
| LCN15 | IGHA2 | FAM3C | MPL | TRDV3 |
| CETP | IGHD | FAM3D | MTNR1A | TRGV9 |
| FABP12 | IGHD1-1 | FGF1 | MTNR1B | TRGV8 |
| FABP9 | IGHD1-14 | FGF11 | NGFR | TRGV5 |
| BPIFA1 | IGHD1-20 | FGF12 | NMBR | TRGV4 |
| LCNL1 | IGHD1-26 | FGF13 | NPR1 | TRGV3 |
| C8G | IGHD1-7 | FGF14 | NPR3 | TRGV2 |
| SPAG11A | IGHD2-15 | FGF16 | NR0B1 | TRGJP2 |
| PI15 | IGHD2-2 | FGF17 | NR0B2 | TRGJP1 |
| NOX1 | IGHD2-21 | FGF18 | NR1D1 | TRGJP |
| PMP2 | IGHD2-8 | FGF19 | NR1D2 | TRGJ2 |
| APOD | IGHD3-10 | FGF20 | NR1H2 | TRGJ1 |
| ORM2 | IGHD3-16 | FGF21 | NR1H3 | TRGC2 |
| ORM1 | IGHD3-22 | FGF22 | NR1H4 | TRGC1 |
| TNF | IGHD3-3 | FGF23 | NR1I2 | TRAV6 |
| CTSG | IGHD3-9 | FGF3 | | |

Table S4. Primers used in this study.

| Gene | Forward primer | Reverse primer |
|---|---|---|
| LDHA | ATGGCAACTCTAAAGGATCAGC | CCAACCCCAACAACTGTAATCT |
| DDIT4 | TGAGGATGAACACTTGTGTGC | CCAACTGGCTAGGCATCAGC |
| ADM | ATGAAGCTGGTTTCCGTCG | GACATCCGCAGTTCCCTCTT |
| ARRB1 | AAAGGGACCCGAGTGTTCAAG | CGTCACATAGACTCTCCGCT |
| ERO1A | GGCTGGGGATTCTTGTTTGG | AGTAACCACTAACCTGGCAGA |
| NAGK | CACGATCCGAGGTCCTTTTAGT | GATCAGCCAGTGGTTTGTGC |
| NRAS | ATGACTGAGTACAAACTGGTGGT | CATGTATTGGTCTCTCATGGCAC |
| NR2F2 | TCATGGGTATCGAGAACATTTGC | TTCAACACAAACAGCTCGCTC |

| | | |
|---|---|---|
| FURIN | CCTGGTTGCTATGGGTGGTAG | AAGTGGTAATAGTCCCCGAAGA |
| β-ACTIN | CATGTACGTTGCTATCCAGGC | CTCCTTAATGTCACGCACGAT |

Table S5. The normalized expression levels of 9 genes.

| ID | ADM | ARRB1 | DDIT4 | ER01A | FURIN | LDHA | NAGK | NR2F2 | NRAS |
|---|---|---|---|---|---|---|---|---|---|
| P1 | 0.152 | 0.207 | 0.530 | 0.007 | 0.005 | 0.063 | 0.016 | 0.010 | 0.549 |
| P2 | 0.033 | 0.187 | 0.153 | 0.005 | 0.007 | 0.090 | 0.005 | 0.015 | 0.397 |
| P3 | 0.664 | 0.306 | 0.053 | 0.016 | 0.017 | 0.136 | 0.017 | 0.018 | 0.187 |
| P4 | 0.028 | 0.123 | 0.037 | 0.012 | 0.007 | 0.104 | 0.016 | 0.025 | 0.088 |
| P5 | 0.147 | 0.169 | 0.059 | 0.051 | 0.082 | 0.008 | 0.047 | 0.056 | 0.102 |
| P6 | 0.010 | 0.052 | 0.030 | 0.023 | 0.016 | 0.126 | 0.013 | 0.038 | 0.077 |
| P7 | 0.026 | 0.071 | 0.082 | 0.016 | 0.018 | 0.150 | 0.014 | 0.043 | 0.199 |
| P8 | 0.019 | 0.047 | 0.054 | 0.012 | 0.010 | 0.142 | 0.029 | 0.042 | 0.262 |
| P9 | 0.030 | 0.176 | 0.140 | 0.006 | 0.007 | 0.094 | 0.011 | 0.049 | 0.061 |
| P10 | 0.015 | 0.083 | 0.009 | 0.024 | 0.017 | 0.371 | 0.028 | 0.030 | 0.108 |
